# Ufmylation on UFBP1 alleviates non-alcoholic fatty liver disease by modulating hepatic endoplasmic reticulum stress

**DOI:** 10.1038/s41419-023-06095-2

**Published:** 2023-09-02

**Authors:** Ziming Mao, Xiaowen Ma, Yu Jing, Minyan Shen, Xirui Ma, Jing Zhu, Huifang Liu, Guangya Zhang, Fengling Chen

**Affiliations:** 1 Department of Endocrinology and Metabolism, Shanghai Ninth People’s Hospital, Shanghai JiaoTong University School of Medicine, Shanghai, 201999 China; 2 School of Graduate, Bengbu Medical College, Bengbu, Anhui 233030 China; 3 Department of Cardiology, Shanghai Sixth People’s Hospital, Shanghai JiaoTong University School of Medicine, Shanghai, 200233 China

**Keywords:** Type 2 diabetes, Non-alcoholic fatty liver disease, Ubiquitylation

## Abstract

Non-alcoholic fatty liver disease (NAFLD) is the most common liver disease characterized by lipid accumulation and endoplasmic reticulum (ER) stress, while effective therapies targeting the specific characteristics of NAFLD are limited. Ufmylation is a newly found post-translational modification process that involves the attachment of the Ubiquitin-fold modifier 1 (UFM1) protein to its substrates via ufmylation modification system. Ufmylation regulates ER stress via modifying UFM1 binding protein 1 (UFBP1), suggesting a potential role for ufmylation in NAFLD pathogenesis. However, the precise role of ufmylation in NAFLD remains unclear. Herein, we aim to elucidate the impact of ufmylation on UFBP1 in NAFLD and explore the underlying mechanisms involved. We observed increased expression of UFM1-conjugated proteins and ufmylation modification system components in livers with steatosis derived from NAFLD patients and NAFLD models. Upregulation of ufmylation on hepatic proteins appeared to be an adaptive response to hepatic ER stress in NAFLD. In vitro, knocking down UFBP1 resulted in increased lipid accumulation and lipogenesis in hepatocytes treated with free fatty acids (FFA), which could be rescued by wild-type UFBP1 (WT UFBP1) but not by a mutant form of UFBP1 lacking the main ufmylation site lys267 (UFBP1 K267R). In vivo, ufmylation on UFBP1 ameliorated obesity, hepatic steatosis, hepatic lipogenesis, dyslipidemia, insulin resistance and liver damage in mice with NAFLD induced by a high fat diet (HFD). We also demonstrated that the downregulation of UFBP1 induced ER stress, whereas the reintroduction or overexpression of UFBP1 alleviated ER stress in a manner dependent on ufmylation in NAFLD. This mechanism could be responsible for the amelioration of aberrant hepatic lipogenesis and insulin resistance in NAFLD. Our data reveal a protective role of ufmylation on UFBP1 against NAFLD and offer a specific target for NAFLD treatment.

## Introduction

Nonalcoholic fatty liver disease (NAFLD) is the most common chronic liver disease comprising a series of nonalcoholic factors-caused liver diseases, ranging from non-alcoholic fatty liver to non-alcoholic steatohepatitis (NASH), fibrosis and cirrhosis [1, 2]. The global prevalence of NAFLD is over 25% and uncontrolled NAFLD can progress to serious pathological lesions such as fatal cirrhosis and hepatocellular carcinoma [3]. However, due to the complexity of NAFLD, approved therapeutic strategies targeting the characteristics of NAFLD are limited [4, 5].

Multiple factors determine NAFLD’s progression. Abnormal lipid metabolism is a crucial phenotype of NAFLD. De novo lipogenesis significantly contributes to the hepatic lipid accumulation in NAFLD [6–8]. Triglyceride (TG) is the main form of hepatic lipid accumulation and cholesterol contributes to NASH development [9, 10]. Meanwhile, elevated serum TG and cholesterol levels are recognized as predictors and promoters of NAFLD [11]. Insulin resistance (IR) is another pivotal phenotype of NAFLD. IR promotes hepatic lipogenesis and lipid deposit in NAFLD [12]. In turn, excessive lipid accumulation exacerbates IR [13]. Additionally, serum alanine aminotransferase (ALT) and aspartate aminotransferase (AST) serve as indicators of hepatocyte damage in NAFLD [14].

ER stress is a prominent feature of NAFLD, which promotes lipogenesis, IR and hepatocyte damage [5, 15]. In NAFLD, hepatic ER stress is triggered by lipid accumulation and activates the unfolded protein response (UPR) [16]. The UPR consists of three pathways: the Inositol-Requiring Enzyme 1α (IRE1α) pathway, the PRKR-Like Endoplasmic Reticulum Kinase (PERK) pathway and the Activating Transcription Factor 6 (ATF6) pathway [17]. Phosphorylated IRE1α induces the expression of X-Box Binding Protein 1 spliced (XBP1s) [18–20] and Caspase 2 [21, 22], which induces hepatic lipogenesis, IR and hepatocyte damage. Similarly, phosphorylated PERK induces the phosphorylation of Eukaryotic Translation Initiation Factor 2 Subunit Alpha (eIF2α) and the expression of Activating Transcription Factor 4 (ATF4), which promotes liver steatosis, IR and hepatocyte damage [5, 23]. Besides, ATF6 is transported to the Golgi apparatus for cleavage during ER stress, but the role of ATF6 in NAFLD remains elusive.

Ufmylation is a new protein modification regulating ER stress [24, 25]. Mature UFM1 is generated from UFM1 precursor through the action of UFM1 Specific Peptidases [26]. Subsequently, UFM1 is transferred to substrates via Ubiquitin Like Modifier Activating Enzyme 5 (UBA5), Ubiquitin-Fold Modifier Conjugating Enzyme 1 (UFC1) and UFM1 Specific Ligase 1 (UFL1) [27, 28]. In ER stress, IRE1α/XBP1 branch promotes the expression of ufmylation modification system components, serving as an adaptive response to ER disturbance [29, 30]. The absence of ufmylation leads to significant ER stress [24, 31–36] and contributes to various diseases, including diabetes [24], ischemic heart disease [37], heart failure [34], hematologic diseases [38, 39], atherosclerosis [40, 41] and tumors [42, 43]. UFBP1 is the first-identified substrate of ufmylation and promotes further ufmylation of other substrates [44], which makes UFBP1 an pivotal factor in ufmylation. Ufmylation on UFBP1 promotes the stability of IRE1α [32], as well as the ER development [29] and ER-autophagy [45], all of these maintain the ER homeostasis. Additionally, UFBP1 is involved in various diseases, including cancers [42, 46, 47], skeletal dysplasia [48, 49], anemia [38, 39] and diabetes [24].

Our previous studies demonstrated that ufmylation prevented ER stress-induced apoptosis and promoted cholesterol efflux in macrophage cells [41, 50]. We also identified Prolyl 4-Hydroxylase Subunit Beta (P4HB) as a new substrate of ufmylation. Defective P4HB ufmylation led to mitochondrial function damage, oxidative stress and ER stress in hepatocytes [35]. In this article, we observe an up-regulated ufmylation of hepatic proteins in NAFLD. We demonstrate that UFBP1 mitigates NAFLD-related phenotypes by regulating hepatic ER stress in an ufmylation-dependent manner. These findings suggest that targeting ufmylation on UFBP1 is a potential therapy for NAFLD.

## Results

### The ufmylation of hepatic proteins is increased in livers with steatosis

**Fig. 1:**
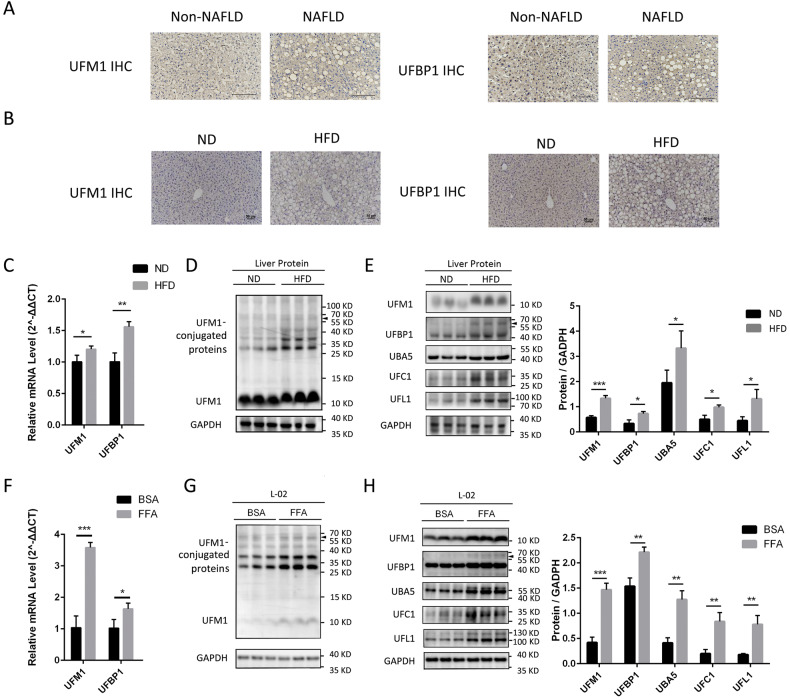
The expression of ufmylation modification system components and UFM1-conjugated proteins is increased in NAFLD. **A** Representative immunohistochemical (IHC) staining of UFM1 and UFBP1 in the liver samples obtained from NAFLD patients or patients without NAFLD (*n* = 7 in each group. Scale bar, 150 μm). **B** Representative IHC staining of UFM1 and UFBP1 in the livers from mice treated with HFD or ND for 12 weeks (*n* = 3 in each group. Scale bar, 50 μm). **C** The mRNA levels of UFM1 and UFBP1 in the livers from mice feeding HFD or ND for 12 weeks (*n* = 3 in each group). **D** The expression of UFM1-conjugated proteins in the livers from mice feeding HFD or ND for 12 weeks (*n* = 3 in each group). Arrow head indicates UFM1-conjugated UFBP1. **E** The expression of ufmylation modification system components (including UFM1, UFBP1, UBA5, UFC1, and UFL1) in the livers from mice feeding HFD or ND for 12 weeks (*n* = 3 in each group). Protein expression was normalized to that of GAPDH. Arrow head indicates UFM1-conjugated UFBP1. **F** The mRNA levels of UFM1 and UFBP1 in L02 cell lines treated with free fatty acids (FFA, OA/PA = 200 μM/100 μM) or vehicle solution (BSA) for 24 h (*n* = 3 in each group). **G** The expression of UFM1-conjugated proteins in L02 cell lines treated with FFA or BSA for 24 h (*n* = 3 in each group). Arrow head indicates UFM1-conjugated UFBP1. **H** The expression of ufmylation modification system components (including UFM1, UFBP1, UBA5, UFC1, and UFL1) in L02 cell lines treated with FFA or BSA for 24 h (*n* = 3 in each group). Protein expression was normalized to that of GAPDH. Arrow head indicates UFM1-conjugated UFBP1. The data in (**C**, **D**, **E**, **F**, **G**, **H)** were presented as the means ± SDs and analyzed by two-tailed Student’s *t* test. *p < 0.05; **p < 0.01; ***p < 0.001.

The expression of UFM1 and its main substrate UFBP1 was assessed in liver samples derived from patients with or without NAFLD using immunohistochemical (IHC) analysis. UFM1 and UFBP1 exhibited higher expression in livers with steatosis compared to non-steatosis samples (Fig. 1A). Similarly, IHC analysis revealed elevated levels of UFM1 and UFBP1 in the livers of mice with high fat diet (HFD)-induced NAFLD in comparison to mice fed a normal chow diet (ND) (Fig. 1B). HFD promoted the transcription of UFM1 and UFBP1 (Fig. 1C) and the expression of UFM1-conjugated proteins compared with ND mice (Fig. 1D). Notably, HFD group exhibited increased protein bands between 55 and 77 KD in both UFM1 and UFBP1 immunoblots, indicating the expression of UFM1-conjugated UFBP1 was promoted by HFD. The enhanced ufmylation of hepatic proteins could be attributed to the increased expression of ufmylation modification system components (UFM1, UBA5, UFC1, UFL1 and UFBP1) in NAFLD (Fig. 1E). Similar results were observed in NAFLD cell models deriving from the immortalized human hepatocyte cell line L02, which were induced by free fatty acids (FFA) (oleate/palmitate, 2:1 ratio) (Fig. 1F–H). Taken together, the ufmylation of hepatic proteins is increased in livers with steatosis, which involves the ufmylation of UFBP1. Based on these results, we aimed to investigate the impact of ufmylation on hepatic proteins in NAFLD, especially the ufmylation on UFBP1. More in-depth results were obtained in the following experiments.

### UFBP1 deficiency promotes FFA-induced hepatocyte steatosis

**Fig. 2:**
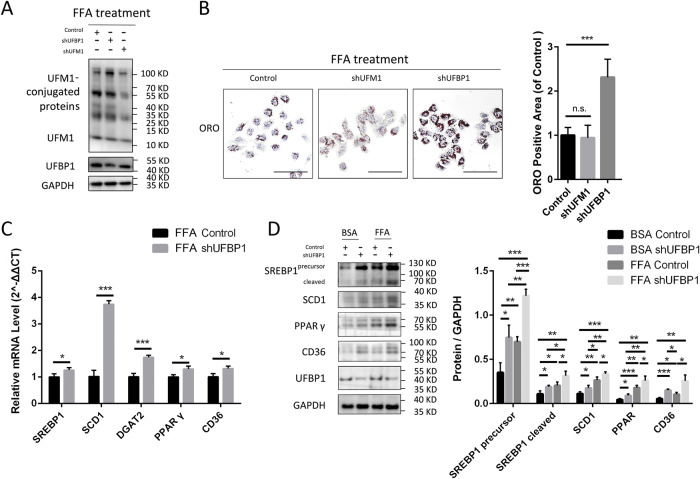
UFBP1 deficiency promotes FFA-induced lipid deposit and hepatic lipogenesis in hepatocytes in vitro. **A** The expression of UFM1 and UFBP1 in L02 cell lines infected with the corresponding control lentivirus-shRNA, lentivirus-shUFM1 or lentivirus-shUFBP1 (Control, shUFM1 and shUFBP1) and treated with FFA (OA/PA = 200 μM/100 μM) for 24 h. **B** Representative images of oil red O (ORO) staining of the indicated L02 cell lines (Control, shUFM1 and shUFBP1) treated with FFA. ORO positive areas were quantified by calculating the ratio of the ORO stained area to the total cell area using Image-Pro Plus and were normalized to those of the control group (*n* = 4 in each group. Scale bar, 50 μm). **C** The mRNA levels of hepatic lipogenic genes (including SREBP1, SCD1, DGAT2, PPARγ and CD36) in the indicated L02 cell lines (Control and shUFBP1) treated with FFA for 24 h (*n* = 3 in each group). **D** The protein levels of SREBP1 (precursor and cleaved forms), SCD1, PPARγ and CD36 in the indicated L02 cell lines treated with FFA or vehicle solution (BSA) for 24 h (*n* = 3 in each group). Protein expression was normalized to that of GAPDH. The data in (**B**, **C** and **D**) were presented as the means ± SDs and analyzed by two-tailed Student’s *t* test. *p < 0.05; **p < 0.01; ***p < 0.001. n.s., non-specific signals.

Hepatocyte steatosis was induced by FFA in L02 cells with UFM1 or UFBP1 knockdown using short hairpin RNA (shRNA). UFBP1 knockdown led to enhanced lipid accumulation in FFA-treated hepatocytes as shown by Oil red O (ORO) staining, whereas UFM1 knockdown didn’t exhibit this effect (Fig. 2A, B). The deficiency of UFBP1 increased the mRNA levels of lipogenic genes, including sterol regulatory element binding protein 1 (SREBP1), stearoylCoA desaturase 1 (SCD1), diacylglycerol O-acyltransferase 2 (DGAT2), peroxisome proliferator-activated receptor γ (PPARγ) and CD36 (Fig. 2C). Consistent with qPCR results, the protein levels of SREBP1 (precursor and cleaved forms), SCD1, PPARγ and CD36 in shUFBP1 group were higher than the control group, regardless of whether or not FFA treatment (Fig. 2D). Collectively, UFBP1 deficiency promotes lipid accumulation and lipogenesis in FFA-induced hepatocyte steatosis.

### Ufmylation on UFBP1 suppresses FFA-induced hepatocyte steatosis

**Fig. 3:**
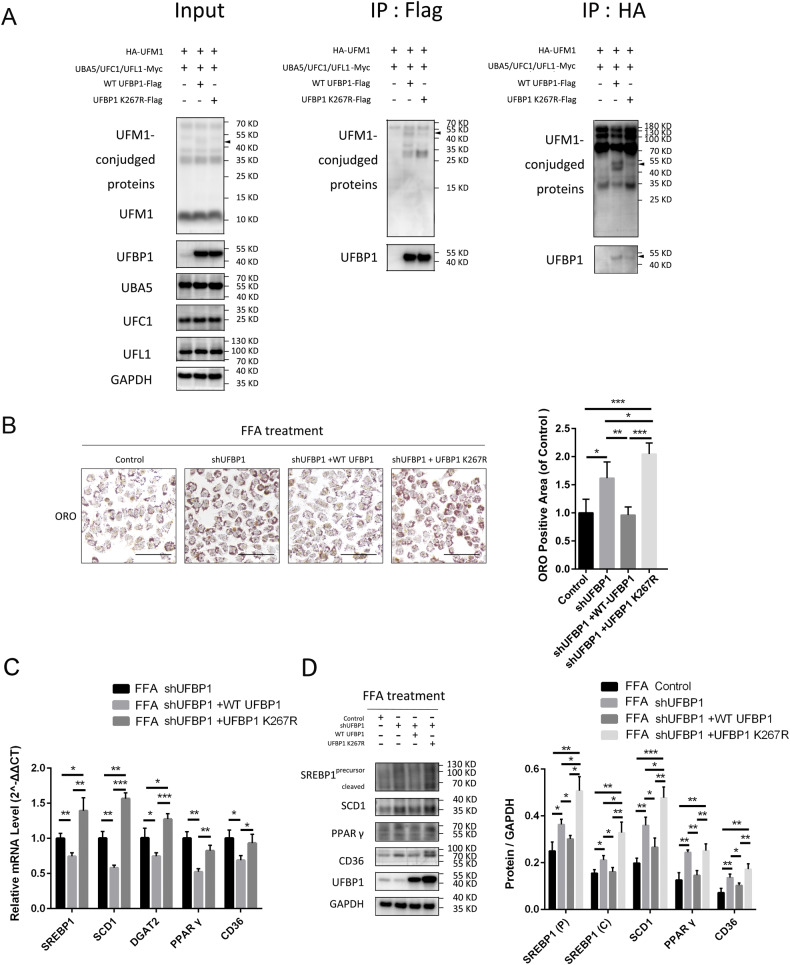
Ufmylation on UFBP1 suppresses lipid accumulation and lipogenesis in FFA-treated hepatocytes in vitro. **A** Analysis of ufmylation on WT UFBP1 and UFBP1 K267R in HEK293T cells expressed the ufmylation system components in various combinations as indicated. Ufmylation of WT UFBP1 and UFBP1 K267R were analyzed by immunoprecipitation with Flag antibody and HA antibody respectively, followed by Western blot with UFBP1 antibody and UFM1 antibody respectively. Arrow head indicates UFM1-conjugated UFBP1. **B** Representative images of oil red (ORO) staining of the indicated L02 cell lines treated with FFA, including cell lines infected with Control lentivirus-shRNA, lentivirus-shUFBP1, and cell lines in which endogenous UFBP1 was knocked down and exogenous WT UFBP1 or UFBP1 K267R was re-expressed (Control, shUFBP1, shUFBP1+WT UFBP1, and shUFBP1 + UFBP1 K267R). All these cell lines were treated with FFA (OA/PA = 200 μM/100 μM) for 24 h. ORO positive areas were quantified by calculating the ratio of the ORO stained area to the total cell area using Image-Pro Plus and were normalized to those of the control group (*n* = 4 in each group. Scale bar, 50 μm). **C** The mRNA levels of hepatic lipogenic genes (SREBP1, SCD1, DGAT2, PPARγ, and CD36) in the indicated L02 cell lines (shUFBP1, shUFBP1+WT UFBP1 and shUFBP1 + UFBP1 K267R) treated with FFA for 24 h (*n* = 3 in each group). **D** The protein levels of SREBP1 (precursor and cleaved forms), SCD1, PPARγ and CD36 in the indicated L02 cell lines (Control, shUFBP1, shUFBP1+WT UFBP1, and shUFBP1 + UFBP1 K267R) treated with FFA for 24 h (*n* = 3 in each group). Protein expression was normalized to that of GAPDH. The data in (**B**, **C** and **D**) were presented as the means ± SDs and analyzed by two- tailed Student’s *t* test. *p < 0.05; **p < 0.01; ***p < 0.001.

The 267 lysine residue (K267) on UFBP1 is the main site for ufmylation, which makes UFBP1 K267R a mutant deficient in ufmylation. To validate this, plasmids encoding wild-type UFBP1 (WT UFBP1)-Flag or UFBP1 K267R-Flag were transfected into HEK293T cells together with plasmids encoding ufmylation modification system components (HA-UFM1, Myc-UBA5, Myc-UFC1, and Myc-UFL1). The cell lysates were subjected to immunoprecipitation using anti-FLAG or anti-HA M2 affinity gel to selectively isolate UFBP1 or UFM1-conjugated proteins. The ufmylation on UFBP1 was significantly decreased when Lys267 was replaced, as shown by protein bands in UFM1 and UFBP1 immunoblots of precipitated proteins (Fig. 3A).

Subsequently, we restored the expression of UFBP1 in UFBP1-knocked down L02 cells with WT UFBP1 or UFBP1 K267R. The promoted FFA-induced lipid accumulation in shUFBP1 group was mitigated by reintroducing WT UFBP1, while reintroducing UFBP1 K267R enhanced the lipid accumulation (Fig. 3B). Re-expressing WT UFBP1 also suppressed the transcription of lipogenic genes (SREBP1, SCD1, DGAT2, PPARγ, and CD36) compared to the shUFBP1 cells under FFA treatment. Conversely, re-expressing UFBP1 K267R promoted the transcription of SREBP1, SCD1 and DGAT2 (Fig. 3C). We also observed that WT UFBP1 suppressed the expression of SREBP1 (precursor and cleaved forms), SCD1, PPARγ and CD36, whereas UFBP1 K267R had no impact on PPARγ and CD36 expression but promoted SREBP1 and SCD1 expression (Fig. 3D). These findings indicate that UFBP1 suppresses FFA-induced hepatocyte steatosis in an ufmylation-dependent manner.

### Ufmylation on UFBP1 facilitates the mitigation of obesity and hepatic steatosis in NAFLD mice

We further validated the protective role of ufmylation on UFBP1 in mice with NAFLD. Due to previous reports of embryonic and adult mouse mortality resulting from UFBP1 knockout [38, 51], UFBP1 knockdown mice were not employed. Instead, adeno-associated virus 8 (AAV8) with high affinity for hepatocytes was used to express either WT UFBP1 or UFBP1 K267R in the livers of mice exposed to ND or HFD (feeding for 12 weeks). Mice fed with ND were injected with AAV-GFP, AAV-WT UFBP1 or AAV-UFBP1 K267R and were euthanized 12 weeks after injection (Supplementary Figure 1A). WB revealed that hepatic UFBP1 expression of ND mice was enhanced by AAV-WT UFBP1 or AAV-UFBP1 K267R injection (Supplementary Fig. 1B). However, ORO staining and Hematoxylin eosin (HE) staining revealed that overexpressing WT UFBP1 or UFBP1 K267R exerted no obvious effect on hepatic lipid accumulation or histopathologic changes in ND mice (Supplementary Fig. 1C).

**Fig. 4:**
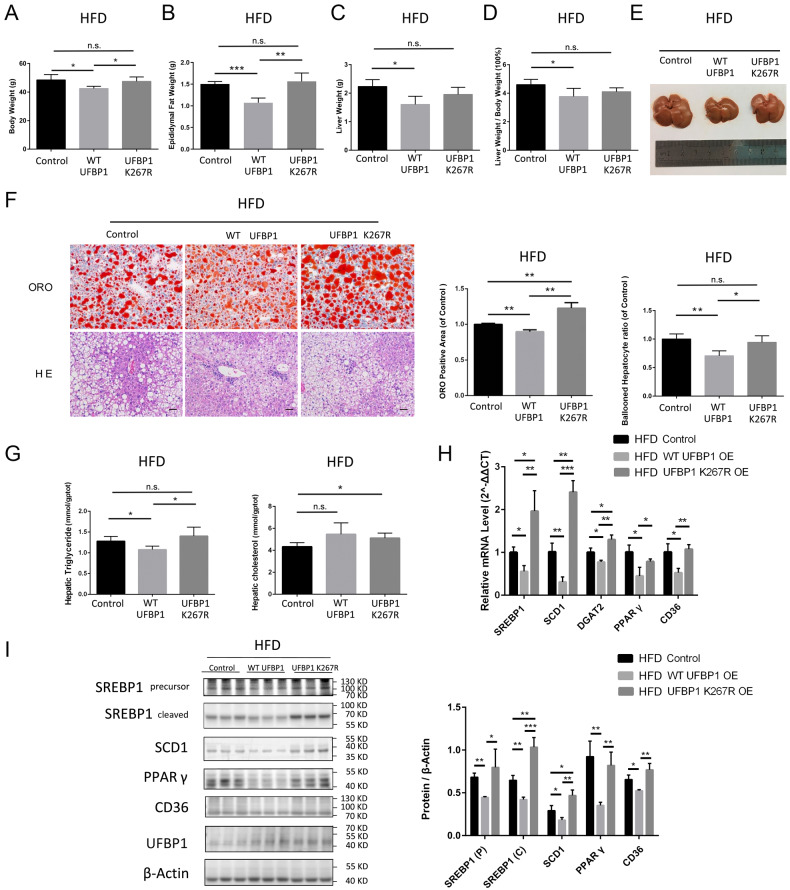
Ufmylation on UFBP1 facilitates the mitigation of obesity and hepatic steatosis and suppresses hepatic lipogenesis in NAFLD mice. **A** The body weight of HFD-fed mice in the indicated groups (mice injected with AAV8-GFP, AAV8-WT UFBP1 or AAV8-UFBP1 K267R) at 12 weeks post-AAV injection (*n* = 4 in each group). **B** The epididymal fat weight of HFD-fed mice in the indicated groups at 12 weeks post-AAV injection (*n* = 4 in each group). **C** The liver weight of HFD-fed mice in the indicated groups at 12 weeks post-AAV injection (*n* = 4 in each group). **D** The ratio of liver weight to body weight (LW/BW) of HFD-fed mice from the indicated groups at 12 weeks post-AAV injection (*n* = 4 in each group). **E** The liver morphology of HFD-fed mice from the indicated groups at 12 weeks post-AAV injection. **F** Representative images of ORO and HE staining of liver sections from HFD-fed mice in the indicated groups at 12 weeks post-AAV injection (*n* = 4 in each group. Scale bar, 50 μm). ORO positive areas were quantified by calculating the ratio of the ORO stained area to the total area of an image using Image-Pro Plus and were normalized to those of the control group. Hepatocyte ballooning ratios were quantified by calculating the ratio of the number of ballooned hepatocytes to the total number of hepatocytes in per high-magnification field and were normalized to those of the control group. **G** Hepatic triglycerides (TG) levels (Left panels) and total cholesterol (TC) (Right panels) levels of HFD-fed mice from the indicated groups at 12 weeks post-AAV injection (*n* = 4 in each group). **H** The mRNA levels of hepatic lipogenic genes (including SREBP1, SCD1, DGAT2, PPARγ and CD36) in HFD-fed mice from the indicated groups at 12 weeks post-AAV injection (*n* = 3 in each group). **I** The protein levels of SREBP1 (precursor and cleaved forms), SCD1, PPARγ and CD36 in the livers of HFD-fed mice from the indicated groups at 12 weeks post-AAV injection (*n* = 3 in each group). Protein expression was normalized to that of β-actin. The data in (**A**, **B**, **C**, **D**, **F**, **G**, **H** and **I**) were presented as the means ± SDs and analyzed by two- tailed Student’s *t* test. *p < 0.05; **p < 0.01; ***p < 0.001. n.s., non-specific signals.

To investigate the influence of ufmylation on UFBP1 in NAFLD, HFD mice with similar body weights were injected with the indicated AAVs to overexpress WT UFBP1 or UFBP1 K267R in livers. These mice were subjected to a prolonged HFD feeding for 12 weeks (Supplementary Fig. 2A, B). At 12 weeks post-AAV injection, the mice in WT UFBP1 group had significantly lower body weight compared to the corresponding controls, while the mice in UFBP1 K267R group had no difference in body weight compared with the control group (Fig. 4A). The decreased body weight in WT UFBP1 group was attributed to the reduced weights of epididymal fat and liver. However, UFBP1 K267R group didn’t exhibit lower weights of epididymal fat or liver compared to the control group (Fig. 4B, C). The ratio of liver weight to body weight (LW/BW) in WT UFBP1 group was also lower than that in the control group, while UFBP1 K267R group didn’t show a decline in LW/BW (Fig. 4D). Furthermore, weight loss in WT UFBP1 group could not be attributed to reduced feeding, as there was no significant difference in caloric intake among the three groups (Supplementary Fig. 2C). The livers of WT UFBP1 group exhibited ruddy color and decreased volume compared to the control group, while the livers of UFBP1 K267R group didn’t show any improvement (Fig. 4E). Furthermore, ORO and HE staining demonstrated that overexpressing WT UFBP1 in livers of HFD mice reduced hepatic lipid accumulation and hepatocellular ballooning compared with the control group. However, overexpressing UFBP1 K267R didn’t alleviate hepatocellular ballooning but promoted lipid accumulation in NAFLD livers (Fig. 4F). Besides, WT UFBP1 overexpression decreased the hepatic TG levels compared to the control group, whereas UFBP1 K267R overexpression didn’t. The total hepatic cholesterol (TC) levels in UFBP1 K267R group were higher than the control group, while the hepatic TC levels in WT UFBP1 group didn’t show statistically significant change (Fig. 4G).

Overexpression of WT UFBP1 in livers of NAFLD mice also suppressed the transcription of lipogenic genes (SREBP1, SCD1, DGAT2, PPARγ, and CD36) in livers compared to the control group. Conversely, overexpression of UFBP1 K267R promoted the transcription of SREBP1, SCD1 and DGAT2 (Fig. 4H). Furthermore, the protein levels of SREBP1 (precursor and cleaved forms), SCD1, PPARγ and CD36 were decreased in livers of WT UFBP1 group compared with the control group, while overexpressing UFBP1 K267R didn’t result in the suppression of PPARγ or CD36, but led to increased expression of SREBP1 and SCD1 (Fig. 4I). These findings suggest that ufmylation on UFBP1 protects against obesity and hepatic steatosis in NAFLD mice.

### Ufmylation on UFBP1 regulates insulin resistance, hypertriglyceridemia and liver damage in NAFLD mice

**Fig. 5:**
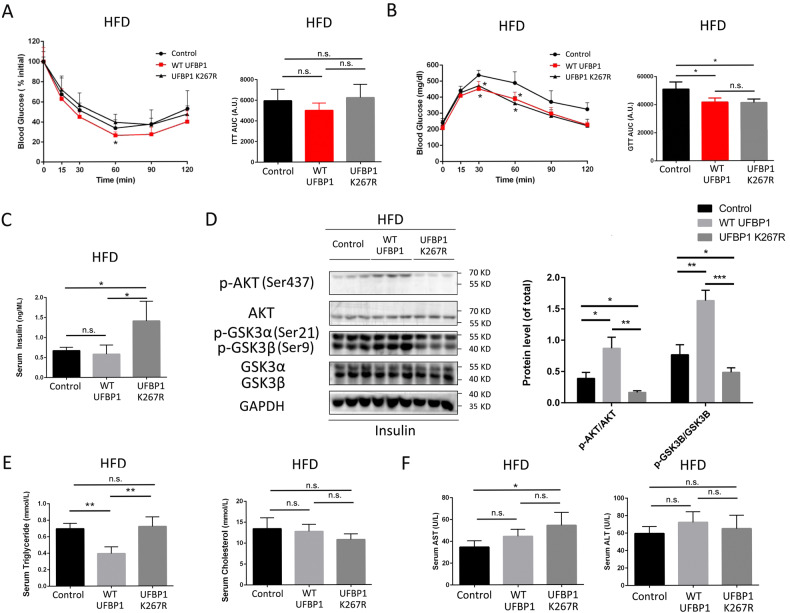
Ufmylation on UFBP1 relieves insulin resistance, hypertriglyceridemia and liver damage in NAFLD mice. **A** Insulin tolerance test (ITT; 0.75 U insulin/kg body weight) on HFD mice from the indicated groups at 11 weeks post-AAV injection. The area under the curve (AUC) of blood glucose level was calculated (*n* = 4 in each group). **B** Glucose tolerance test (GTT; 1 g glucose/kg body weight) on HFD mice from the indicated groups at 10 weeks post-AAV injection. The area under the curve (AUC) of blood glucose was calculated (*n* = 4 in each group). **C** Fasting serum insulin levels of HFD mice from the indicated groups at 12 weeks post-AAV injection (*n* = 4 in each group). **D** The phosphorylation levels of AKT and GSK3β in the livers of HFD mice from the indicated groups at 12 weeks post-AAV injection (*n* = 3 in each group). Mice received intraperitoneal insulin injection (0.75 U/kg) 10 min before liver tissue collection. Phosphorylation levels were normalized to the level of total proteins (*n* = 3 in each group). **E** Fasting serum triglycerides (TG) (Left panels) and fasting serum total cholesterol (TC) (Right panels) of HFD mice from the indicated groups at 12 weeks post-AAV injection (*n* = 4 in each group). **F** Serum aspartate aminotransferase (AST) (Left panels) or alanine aminotransferase (ALT) (Right panels) levels of HFD mice from the indicated groups at 12 weeks post-AAV injection (*n* = 4 in each group). The data in (**A**, **B**, **C**, **D**, **E** and **F**) were presented as the means ± SDs and analyzed by two- tailed Student’s *t* test. *p < 0.05; **p < 0.01; ***p < 0.001. A.U. arbitrary units. n.s. non-specific signals.

We then examined the effect of ufmylation of UFBP1 on regulating the impaired glucose homeostasis in NAFLD mice. Glucose tolerance test (GTT) were conducted 10 weeks after the indicated AAVs injection, followed by insulin tolerance test (ITT) a week later. ITT revealed that insulin resistance was ameliorated in WT UFBP1 group compared with the control group. However, this improvement was not observed in UFBP1 K267R group (Fig. 5A). GTT revealed that mice of WT UFBP1 group and UFBP1 K267R group exhibited increased glucose tolerance compared to the control group (Fig. 5B). In addition, fasting serum insulin levels were increased in UFBP1 K267R group but not in WT UFBP1 group (Fig. 5C). Moreover, hepatic insulin signaling was assessed after intraperitoneal injection of insulin. Activation of the insulin signaling pathway was facilitated in livers of WT UFBP1 group compared to the control group, as measured by levels of phosphorylated AKT and phosphorylated glycogen synthase kinase-3β (GSK3β). However, the insulin signaling pathway was suppressed in livers of UFBP1 K267R group (Fig. 5D). These data indicated that ufmylation on UFBP1 improved the insulin sensitivity of NAFLD mice.

Meanwhile, we tested the serum TG and TC levels in the indicated groups at 12 weeks post-injection. The serum TG levels in WT UFBP1 group were lower than the control group, but there was no significant difference in TG levels between UFBP1 K267R group and the control group. Additionally, the levels of serum TC showed no significant difference among the three groups (Fig. 5E). Meanwhile, overexpressing UFBP1 K267R increased the serum AST levels in NAFLD mice, while overexpressing WT UFBP1 didn’t affect the serum aminotransferase levels (Fig. 5F). In general, these tests indicate that ufmylation on UFBP1 regulates insulin resistance, hypertriglyceridemia and liver damage in mice with NAFLD.

### UFBP1 suppresses hepatic ER stress in an ufmylation-dependent way in NAFLD

Given the significance of hepatic ER stress in NAFLD [21] and the role of UFBP1 in maintaining ER homeostasis through ufmylation [32], we investigated the UPR pathways in indicated cell lines and livers of HFD mice injected with AAVs to uncover the underlying mechanism behind the protective effects of ufmylation on UFBP1 in NAFLD.

**Fig. 6:**
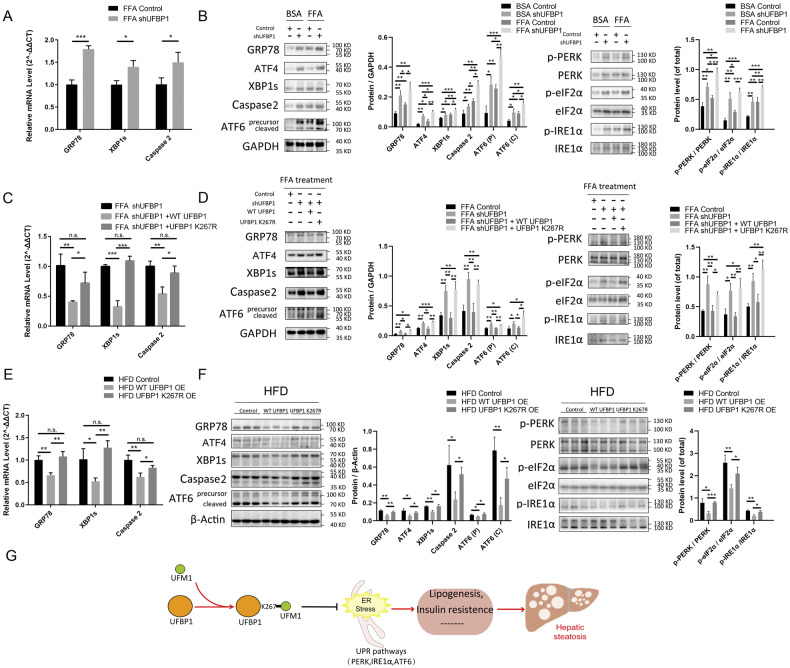
UFBP1 suppresses hepatic ER stress in an ufmylation-dependent way. **A** The mRNA levels of ER stress-related genes (including GRP78, XBP1s and Caspase 2) in the indicated L02 cell lines (Control, shUFBP1) treated with FFA for 24 h (*n* = 3 in each group). **B** The protein levels of GRP78, ATF4, XBP1s, Caspase 2, and ATF6 (precursor and cleaved forms) (Left panels) and phosphorylation levels of PERK, eIF2α and IRE1α (Right panels) in the indicated L02 cell lines treated with FFA or vehicle solution (BSA) for 24 h. Protein expression was normalized to that of GAPDH and phosphorylation levels were normalized to the level of total proteins (*n* = 3 in each group). **C** The mRNA levels of ER stress-related genes (including GRP78, XBP1s and Caspase 2) in the indicated L02 cell lines (Control, shUFBP1, shUFBP1+WT UFBP1, and shUFBP1 + UFBP1 K267R) treated with FFA for 24 h (*n* = 3 in each group). **D** The protein levels of GRP78, ATF4, XBP1s, Caspase 2, and ATF6 (precursor and cleaved forms) (Left panels) and phosphorylation levels of PERK, eIF2α and IRE1α (Right panels) in the indicated L02 cell lines treated with FFA for 24 h. Protein expression was normalized to that of GAPDH and phosphorylation levels were normalized to the level of total proteins (*n* = 3 in each group). **E** The mRNA levels of ER stress-related genes (including GRP78, XBP1s and Caspase 2) in the livers of HFD mice from the indicated groups at 12 weeks post-AAV injection (*n* = 3 in each group). **F** The protein levels of GRP78, ATF4, XBP1s, Caspase 2 and ATF6 (precursor and cleaved forms) (Left panels) and phosphorylation levels of PERK, eIF2α and IRE1α (Right panels) in the livers of HFD mice from the indicated groups at 12 weeks post-AAV injection (*n* = 3 in each group). Protein expression was normalized to that of β-actin and phosphorylation levels were normalized to the level of total proteins. **G** A schematic model depicting that ufmylation on UFBP1 K267 mitigates ER stress and the progression of hepatic steatosis. The data in (**A**, **B**, **C**, **D**, **E**, **F**) were presented as the means ± SDs and analyzed by two- tailed Student’s *t* test. *p < 0.05; **p < 0.01; ***p < 0.001. n.s., non-specific signals.

The transcription of ER stress-related genes (GRP78, XBP1s, and Caspase 2) was promoted in L02 cells with UFBP1 knockdown under FFA treatment (Fig. 6A). UFBP1 deficiency also induced the phosphorylation of ER stress markers (PERK, eIF2α and IRE1α) and the expression of ER stress-related proteins (GRP78, XBP1s, ATF4, ATF6 and Caspase 2) (Fig. 6B). Notably, reintroducing WT UFBP1 into UFBP1-knocked down cells attenuated the ER stress under FFA treatment, while reintroducing UFBP1 K267R did not relieve ER stress in shUFBP1 cells (Fig. 6C, D). Moreover, the transcription of ER stress-related genes was decreased in livers of WT UFBP1 group compared to the control group, which was not observed in UFBP1 K267R group (Fig. 6E). Consistent with qPCR results, overexpressing WT UFBP1 but not UFBP1 K267R suppressed the phosphorylation and expression of ER stress markers in NAFLD livers (Fig. 6F).

In conclusion, our study provides evidence that ufmylation on UFBP1 is a novel player in NAFLD. Ufmylation on UFBP1 is upregulated in the livers with steatosis, which in turn relieves hepatic ER stress. The inhibition of UPR pathways (PERK, IRE1α and ATF6 pathway) suppresses hepatic lipogenesis and insulin resistance, leading to the remission of NAFLD-related phenotypes (Fig. 6G).

## Discussion

In this study, we report the discovery that ufmylation plays a role in mitigating NAFLD for the first time. We observed that the expression of UFM1-conjugated proteins and ufmylation modification system components was elevated in NAFLD, which involved the ufmylation on UFBP1. Furthermore, we demonstrated that ufmylation on UFBP1 K267 was crucial for ameliorating NAFLD phenotypes, including obesity, liver steatosis, hepatic lipogenesis, dyslipidemia, insulin resistance and liver damage. We provide evidence for the protective effects of ufmylation in NAFLD and contribute to the establishment of ufmylation research as a new field for NAFLD treatment.

Ufmylation is found to closely associate with various diseases, including diabetes [24], ischemic heart disease [37], heart failure [34], hematologic diseases [38, 39], atherosclerosis [40, 41] and tumors [42, 43] since its initial discovery in 2004. However, only a few substrates, such as UFBP1 [24], Activating signal cointegrator 1 [44], P53 [42], Meiotic Recombination 11 Homolog 1 [52], Ribosomal Protein L26 (RPL26) [53], Ribophorin I (RPN1) [31], and Histone H4 [54], have been identified so far. In our recent study, we identified P4HB as an ufmylation substrate regulating mitochondrial function damage, oxidative stress and ER stress [35]. Meanwhile, UFBP1 is the first-discovered substrate of ufmylation, which is composed by a signal peptide, a nuclear localization signal, a DDRGK domain and a PCI domain. The signal peptide localizes UFBP1 to ER and the Lys residue 267 within the PCI domain undergoes ufmylation to modulate ER homeostasis [29, 32, 44]. In this study, we further investigated the biological function of ufmylation on UFBP1 and elucidated the precise regulatory mechanism. Our findings suggest that ufmylation on UFBP1 alleviates NAFLD-related symptoms, including obesity, hepatic steatosis, insulin resistance, dyslipidemia and liver damage (Figs. 3–5). It is the first time ufmylation has been identified as a mechanism for ameliorating NAFLD through UFBP1.

We observed that UFBP1 deficiency in hepatocytes promoted lipogenesis, which was reversed by WT UFBP1 but not by UFBP1 K267R in vitro. Furthermore, ufmylation on UFBP1 inhibited hepatic lipogenesis in NAFLD mice. This inhibitory effect was achieved by suppressing the expression of lipogenesis-related nuclear transcription factors (SREBP1 and PPARγ) and important lipogenic genes (SCD1, DGAT2, and CD36). Increased hepatic lipogenesis is a prominent characteristic of NAFLD regulated by an elaborate network [8]. SREBP1 plays a vital role in hepatic lipogenesis and SCD1 is a determinant in triglyceride biosynthesis induced by SREBP1 [55–57], both of which lead to liver steatosis. Additionally, DGAT2 promotes the development of dyslipidemia and NAFLD. Inhibiting DGAT2 reduces hepatic and plasma triglyceride levels in rats fed a Western-type diet [58]. PPARγ is another transcription factor participating in NAFLD [57]. PPARγ regulates the expression of CD36 which promotes lipid acid uptake and contributes to the NAFLD progression. Hepatocyte-specific deletion of CD36 reduces hepatic lipid content and improves insulin sensitivity, thereby mitigating NAFLD [59]. Notably, considering the pivotal role of PPARγ in governing glucose and lipid metabolism, our finding that ufmylation on UFBP1 downregulated PPARγ expression may decipher the phenotypic alterations in hepatic steatosis, serum lipids and insulin sensitivity. Therefore, our research also offers novel insights into the treatment of diabetes.

Mechanistically, we verify that UFBP1 mitigates ER stress in NAFLD through an ufmylation-dependent mechanism. The efficacy of ufmylation in suppressing ER stress through UFBP1 has been widely established in previous studies. Liu et al. reported that ufmylation on UFBP1 K267 up-regulated the stability of IRE1α by interacting with the kinase domain of IRE1α, which suppressed ER stress and apoptotic cell death in HepG2 cells [32]. Additionally, ER-resident ufmylation facilitates the ER-autophagy and the degradation of translation-arrested ER polypeptides, both of which maintain the ER homeostasis [31, 45, 53, 60]. UFBP1 recruits the ufmylation machinery to the surface of ER to promote ufmylation of RPN1, RPL26, and NADH-cytochrome b5 reductase 3 (CYB5R3), which regulates ER-autophagy and mitigates ER stress. Notably, UFBP1’s capacity to recruit the ufmylation machinery to ER is not dependent upon its primary site of ufmylation at K267 [31], whereas ufmylation on UFBP1 K267 is required for ufmylation of CYB5R3, which serves as a signal for ER-autophagy [45]. Meanwhile, UFBP1 recruits UFL1 to the ER membrane and promotes ufmylation on RPL26 [60]. RPL26 ufmylation facilitates the transport of arrested polypeptides from ER to lysosomes for degradation, thereby preventing ER stress [53]. However, it remains obscure whether ufmylation on UFBP1 is involved in RPL26 ufmylation. Not completely the same with previous studies, we didn’t observe significant alteration in IRE1α levels upon UFBP1 knockdown or overexpression. Instead, UFBP1 deficiency led to the activation of all three UPR pathways (IRE1α, PERK, and ATF6 pathway) in hepatocytes, which could be rescued by UFBP1 in an ufmylation-dependent manner. Meanwhile, further investigation is warranted into the effects of ufmylation on UFBP1 in ER-autophagy and ER-arrested polypeptides degradation in livers with NAFLD.

ER stress is a significant pathophysiological characteristic of NAFLD [5, 16]. In ER stress, Caspase 2 is induced by phosphorylated IRE1α to promote the maturation of SREBP1 in NAFLD livers [21]. IRE1α/XBP1s also promotes hepatic lipogenesis by inducing the transcription of lipogenic genes (SREBP1, SCD1, DGAT2 and ACC2) [18]. Meanwhile, activation of IRE1α-mediated XBP1s and JUN N-Terminal Kinase induces hepatic insulin resistance in NAFLD [61]. In addition, Oyadomari et al. discovered that PERK pathway promoted NAFLD via eIF2α and ATF4 [62]. ATF4 depletion protects mice against liver steatosis and hypertriglyceridemia in response to high fructose feeding [23]. ER stress also leads to hepatocyte death and liver damage in NAFLD through the IRE1α pathway and PERK pathway [5, 63–66]. Considering the effectiveness of inhibiting ER stress in treating NAFLD, the conclusion that ufmylation on UFBP1 alleviates NAFLD phenotypes via attenuating hepatic ER stress is reliable. Notably, ER stress plays a role in other liver diseases, including viral hepatitis and hepatocellular carcinoma [16, 67]. Our founding provides a potential target not only for treating NAFLD but also for other ER stress-associated liver diseases.

NAFLD is a prevalent liver disease possessing significant challenges in targeted therapeutic interventions. In this study, we identify novel roles of ufmylation on UFBP1 in ameliorating NAFLD by alleviating hepatic ER stress. Meanwhile, further investigations are required to fully elucidate the precise mechanisms underlying the regulation of ER stress by ufmylation on UFBP1. Moreover, it is warranted to explore the small-molecule drugs that can enhance the ufmylation on UFBP1, such as protein post-translational modification targeting chimera [68], for the therapeutic management of NAFLD.

## Methods

### Animals and treatments

8-week-old male C57BL/6J mice (20–30 g) were purchased from Shanghai JieSiJie Laboratory Animal Co., Ltd., which were raise and kept in a standard environment with a 12-h light/12-h dark cycle and a temperature-controlled environment (22–24 °C). To established mice with hepatic steatosis, mice were fed with a high-fat diet (18.1% protein, 20.3% carbohydrates, 61.6% fat; D12492, Research Diets, New Brunswick, NJ, USA) for 12 weeks, while the corresponding control mice were fed a normal chow diet (18.3% protein, 71.5% carbohydrates, 10.2% fat; D12450B, Research Diets, New Brunswick, NJ, USA). After 12 weeks feeding, the ND mice (*n* = 3) and HFD mice (*n* = 3) were euthanized with liver samples collected. AAV 2/8, a virus known for its high affinity for hepatocytes, were acquired from Hanbio Biotechnology Co., Ltd., Shanghai, China. Recombinant AAV 2/8 were generated with amplification primers: m UFBP1 F 5′-CggatccataggtaccactgccaccATGGACTACAAGGATGA CGATGACAAGGATTACAAAGACGACGA-3’, m UFBP1 R 5′-ccgacatatgtacgatatcgtggaattcTC AGGCTGAAGCCTGGGCAG-3′, and mutant primers : m UFBP1 mutation F 5′-ACTATAAGG ATGATGACGACAAAGTGGGGCCCTGGGTGTATCTGGTGG-3′, m UFBP1 mutation R 5′-TCTGG GGTTATGTAGATAAACcTGCCCCGGTCGTCAATCACACCTGT-3′. Mice fed with a normal chow diet (*n* = 12) were divided into three groups and injected with empty adeno-associated virus 2/8 (HBAAV 2/8-CMV-GFP), recombinant AAV 2/8 expressing wild-type mice UFBP1 and recombinant AAV 2/8 expressing UFBP1 K267R via the tail vein (10^11 vg/mouse), respectively. At 12 weeks post-AAV injection, mice were euthanized and liver samples were collected. Similarly, mice that fed with a 12 weeks high-fat diet and had similar weight (*n* = 12) were divided into three groups, then injected with HBAAV 2/8-CMV-GFP, HBAAV 2/8-CMV-WT UFBP1 and HBAAV 2/8-CMV-UFBP1 K267R via the tail vein (10^11 vg/mouse), respectively. All three groups of HFD mice were treated with a prolonged HFD feeding for 12 weeks and then were euthanized with liver, epididymal fat and serum samples collected. Caloric intake in indicated HFD groups was calculated from food intake of mice at 8 weeks post-AAV injection. All experimental procedures comply with ethical regulations and were approved by the ethics committee of Shanghai Ninth People’s Hospital (approval number SH9H-2021-A608-SB).

### Mouse metabolic analysis

Mice that fed with a 12 weeks high-fat diet (*n* = 12) to established mice with hepatic steatosis were divided into three groups, then injected with AAVs indicated above. To perform the glucose tolerance tests (GTTs) at 10 weeks post-AAV injection, mice were intraperitoneally injected with 1 g glucose/kg body weight after 12 h fasting. Blood glucose level was measured with Blood Glucose Meter (OneTouch Ultra, Johnson Medical Equipment and Materials Company, China) at 0, 15, 30, 60, 90, and 120 min after the glucose injection. To perform ITTs at 11 weeks post-AAV injection, 0.75 U insulin/kg body weight was injected into abdominal cavity of mice after 5 h fasting. Blood glucose levels were measured at 0, 15, 30, 60, 90, and 120 min after the insulin injection. To explore the activation of the insulin signaling pathway in livers at 12 weeks post-AAV injection, mice received intraperitoneal insulin (0.75 U/kg) injection. 10 min after the insulin injection, liver tissues of these mice were collected.

### Mouse serum biochemical examination of TG, TC, AST, ALT, and insulin

Mice serum were obtained after 12 h fasting. The levels of serum TG, TC, ALT and AST were examined by corresponding testing kits purchased from Nanjing Jiancheng Bioengineering Institute, China (Triglycerides Assay Kit, F001-1-1; Total cholesterol Assay Kit, F002-1-1; Alanine aminotransferase Assay Kit, C009-3-1; Aspartate aminotransferase Assay Kit, C010-3-1). The serum levels of insulin were also examined by Ultra Sensitive Mouse Insulin ELISA Kit (90080, CrystaLChem, United States).

### Mouse hepatic triglyceride and cholesterol analysis

Hepatic lipids were extracted from livers of the indicated HFD mice. TG and TC levels in livers were determined using Triglyceride Colorimetric Assay Kit and Cholesterol Quantification Kit mentioned above.

### Histology and immunohistochemical procedures

Liver tissues were fixed with 10% (vol/vol) neutral buffered formalin for 48 h. Then, samples were embedded in paraffin and sliced into 4 μm thick sections. Hematoxylin and eosin (HE) staining was performed on paraffin sections to observe the distribution of lipid accumulation and the hepatocellular ballooning with a HE staining kit (G1003, servicebio, Wuhan, China) according to a standard protocol. For immunohistochemical staining, sections were deparaffinized and rehydrated with xylene and ethanol respectively. 3% hydrogen peroxide was used to inactivate endogenous peroxidase. Sections were blocked with normal goat serum and incubated with primary and secondary antibodies (Primary antibodys to UFM1 (A15843, 1:100; abclonal, Wuhan, China), UFBP1 (21445-1-AP, 1:300, Proteintech, United States) and goat anti-rabbit secondary antibody (RS0002, ImmunoWay Biotechnology, United States)), followed by color development with an DAB Histochemical Kit (G1211, servicebio, Wuhan, China). Immunostaining images were taken with the Nikon Fluorescence Microscope.

### Tissue and cell Oil Red O (ORO) staining

To visualize lipid droplets in the liver, frozen liver tissues from anesthetized mice were embedded in Tissue-Tek OCT compound, and then sliced to 8–10 μm thick frozen sections. Frozen sections were fixed with fixative (G1101, servicebio, Wuhan, China) and stained with Oil Red O (G1016, servicebio, Wuhan, China) without light for 8–10 min. Then the tissue sections were washed with 60% isopropyl alcohol (#I9030, Sigma-Aldrich) and re-stained with hematoxylin. The slides were sealed with glycerin-glutin. For cell ORO staining, cells were wash by PBS (SH30256.01, HyClone, United States) and fixed with 4% paraformaldehyde, then cells were stained with ORO as described above. Images were taken with the Nikon Fluorescence Microscope.

### Human liver samples

Human liver tissues (seven NAFLD subjects and seven non-NAFLD subjects) were collected from patients with hepatocarcinoma or hepatic metastases via clinical hepatic surgery, patients’ consent and authorization were signed before the operation. Patients with excessive alcoholic intake (>140 g for men or >70 g for women, per week), or known history of drug induced liver injury and hepatitis virus infections were excluded from this study. NAFLD or non-NAFLD liver samples were identified by the department of clinical laboratory through HE staining. All procedures involved human sample collection were consistent with the principles in the Declaration of Helsinki and were approved by the ethics committee of Shanghai Ninth People’s Hospital (approval number: SH9H-2021-TK315-1).

### Cell lines and culture

Human L02 hepatocyte cell line and HEK293T cell line were purchased from Chinese Academy of Sciences. L02 cells were cultured in RPMI-1640 (R8758, Nanjing KeyGen Biotech. Co., LTD.) and HEK293T cells were cultured in DMEM (D0822, Sigma-Aldrich), both of which were supplemented with 10% fetal bovine serum (C0227, Beyotime Biotechnology, Shanghai, China) and 1% penicillin–streptomycin (15140-122, Gibco, Carlsbad, CA) in a 5% CO_2_ incubator. To induce lipid accumulation in hepatocytes in vitro, L02 cells were treated with cell culture medium containing free fatty acids (FFA). Palmitic acid (PA; 0.1 mM; Sigma-Aldrich) and oleic acid (OA; 0.2 mM; Sigma-Aldrich) mixture (dissolved in fatty acid–free BSA) was added to the medium for 24 h to establish an in vitro model of lipid accumulation in hepatocytes. The L02 cells treated with fatty acid-free bovine serum albumin (BSA; A602448-0050; BBI Life Sciences, Shanghai, China) were used as control.

### Knockdown of UFM1 or UFBP1 in human L02 hepatocyte cell line

Lentiviral vectors expressing specific shRNAs were constructed using lentiviral knockdown plasmid pLKO.1 system. Specific oligonucleotides were synthesized (BioSune Biotechnology Co., Ltd., Shanghai, China), annealed and cloned into the pLKO.1 plasmid. Lentiviruses were produced by transient transfection of recombinant pLKO.1 plasmid, psPAX2 plasmid and pMD2.G plasmid on HEK293T cell line at a ratio of 3:2:1. Lentiviruses were harvested and concentrated, which were used to infect L02 cell line. Cells that stably expressing shRNA were selected in RPMI-1640 medium supplemented with puromycin (2 μg/ml). The primer sequences used in construction of lentiviruses are listed: h-UFM1 shRNA F 5′-CCGGCAATGATGGAATAGGAATAAACTCGAGTTTATTCCTATTCCATCATTGTTTTTG-3′, h- UFM1 shRNA R 5′-AATTCAAAAACAATGATGGAATAGGAATAAACTCGAGTTTATT CCTATTCCATCATTG-3′; h-UFBP1 shRNA F 5′-CCGGAAGGCGTAGGAGAGACCATGACTCGAG TCATGGTCTCTCCTACGCCTTTTTTTG-3′, h-UFBP1 shRNA R AATTCAAAAAAAGGCGTAGG AGAGACCATGACTCGAGTCATGGTCTCTCCTACGCCTT.

### Co-immunoprecipitation (Co-IP) assay

HEK293T cells were transiently transfected with plasmids encoding Flag-tagged UFBP1 (WT UFBP1-Flag or UFBP1 K267R-Flag) together with plasmids encoding HA-tagged or Myc-tagged ufmylation modification system components (HA-UFM1, Myc-UBA5, Myc-UFC1 and Myc-UFL1) for 48 h. Cells were then solubilized on ice for 30 min in lysis buffer containing 20 mM Tris/HCl (pH 7.6), 150 mM NaCl, 1% Triton X-100, 1% Protease inhibitor (04693116001, Roche) and 1% Phosphatase inhibitors (4906845001, Roche) and were cleared by centrifugation at 18.000 *g* for 20 min, 4 °C. The supernatant was divided into two parts, which were incubated with anti-FLAG M2 Agarose Beads (A2220, Sigma Aldrich, USA) or anti-HA M2 Agarose Beads (AE059, abclonal, Wuhan, China) overnight at 4 °C. Precipitates were obtained by centrifugation at 9.000 *g* and were washed with lysis buffer for three times. Precipitates were boiled in SDS-sampling buffer and subjected to SDS-PAGE followed by immunoblot analysis.

### Restoring UFBP1 in L02 cell line with UFBP1 knockdown

L02 cells with UFBP1 knockdown were transiently transfected with plasmids expressing either WT UFBP1 or UFBP1 K267R to restoring UFBP1. pEGFP N2 plasmids were used to load WT UFBP1 or UFBP1 K267R. Neomycin selection (400 μg/ml) for positively-transduced cells was performed 48 h post transduction for 7 days.

### Quantitative real-time PCR

Tissue samples or cells were lysed in TRIzol (Invitrogen) for RNA isolation according to the manufacturer’s instructions. 1 μg of RNA was used for reverse transcription by PrimeScript™ RT reagent Kit (Perfect Real Time) (RR037A, Takara Bio Inc., Japan) to obtain complementary DNA. Quantitative PCR assays were performed with TB Green® Premix Ex Taq™ (Tli RNaseH Plus) Kit (RR420A, Takara Bio Inc., Japan) in a real-time PCR system (LightCycler 480 Instrument II; Roche Diagnostics Inc., Basel, Switzerland), and mRNA levels were assessed by the comparative cycle threshold method(2−ΔΔCt). The primer information is listed in Supplementary Table 1.

### Western blot analysis

Tissues and cells were lysed with RIPA Lysis Buffer (KGP702-100, KeyGen Biotechnology Co., Ltd., Jiangsu, China) containing 1% PMSF (A100754, Sangon Biotech, Shanghai, China), 1% Protease inhibitor (04693116001, Roche) and 1% Phosphatase inhibitors (4906845001, Roche). Protein concentrations were quantified with a bicinchoninic acid protein assay kit (23225; Thermo Fisher, Rockford, IL). Proteins were separation by 10% SDS-PAGE and transferred to polyvinylidene fluoride, which was then blocked in 5% BSA for 1.5 h and incubated with specific primary antibodies overnight. Related antibodies were purchased from abclonal (Wuhan, China, including UFM1 (A15843), SREBF1 (A15586), SCD1 (A16429), PERK (A18196) and ATF6 (A0202)), Proteintech (United States, including UFBP1 (21445-1-AP) and PPARγ (16643-1-AP)), Abcam (UK, including UBA5 (ab177478), UFC1 (ab189252), UFL1 (ab226216), and UFSP2 (ab185965)), NovusBio (United States, including Phospho-IRE1α (Ser724) (NB100-2323)) and Cell Signaling Technology (United States, including Phospho-PERK (Thr980) (#3179), IRE1α (#3294), eIF2α (#5324), Phospho-eIF2α (Ser51) (#3398), XBP1s (#27901), β-Actin (#4970), GAPDH (#5174)). Secondary antibody with a horseradish peroxidase-labeled, including goat antibody to rat (#7074) or mouse (#7076), were purchased from Cell Signaling Technology (United States) and used to bind to primary antibodies for 1 h. Finally, signals were detected with an enhanced chemiluminescence kit and ChemiDoc MP Imaging System.

### Statistical analyses

For data showing normal distribution, a two-tailed Student’s *t* test was conducted to compare differences between two groups. For data showing a skewed distribution, a nonparametric statistical analysis was performed using the Mann-Whitney *U* test for two-group comparison. All values are shown as means ± SDs. *P* values were categorized as follows: *p < 0.05, **p < 0.01, ***p < 0.001 and a *p* value of <0.05 indicated statistical significance. GraphPad Prism 8.0 software was used to carry out statistical analysis.

## Supplementary information

Supplementary Figure 1

Supplementary Figure 2

Supplementary table_1

Original western blots

A reproducibility checklist

## Data Availability

Supplementary figure 1, 2 and Supplementary table 1 is available as a Supplementary Information file. All data supporting the findings of this study are available from the corresponding authors on reasonable request.
