# Supplementary material for: Ufmylation on UFBP1 alleviates non-alcoholic fatty liver disease by modulating hepatic endoplasmic reticulum stress
Source: Cell Death Dis. 2023 Sep 2;14(9):584. doi: 10.1038/s41419-023-06095-2 (PMC10475044; doi:10.1038/s41419-023-06095-2)
Supplement: Supplementary file 1 — Supplementary Figure 1 [file 41419_2023_6095_MOESM1_ESM.docx]

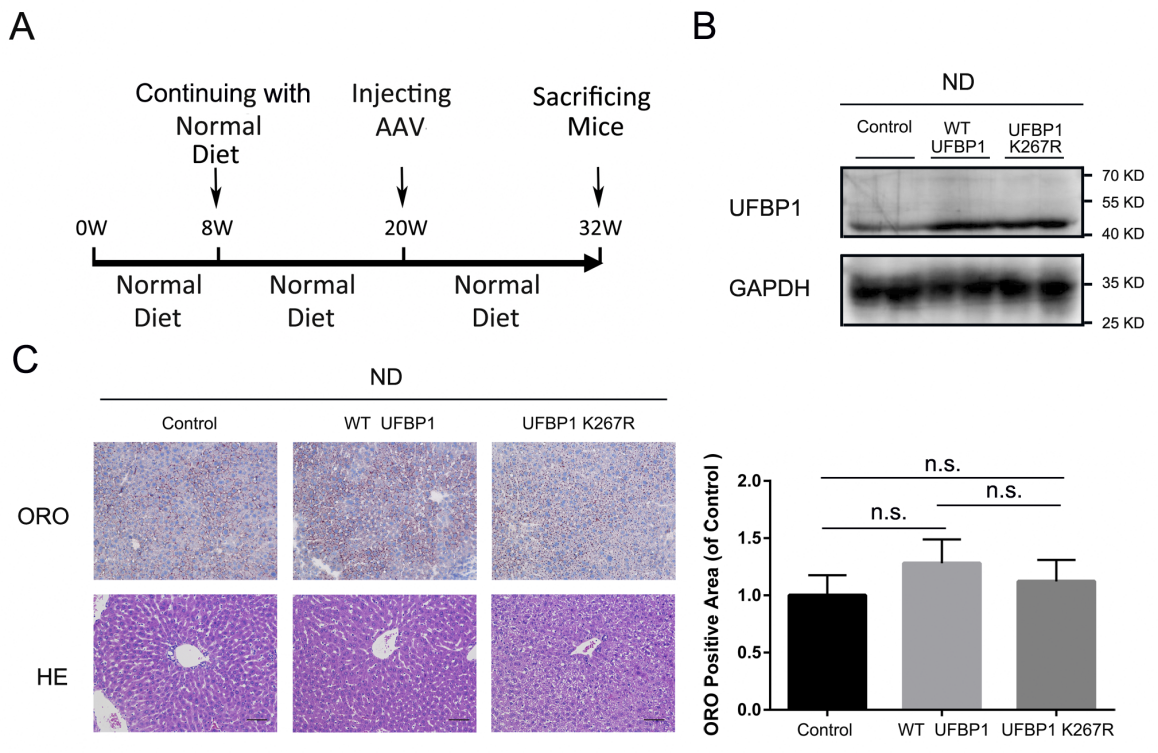


**Supplementary figure 1. Overexpressing exogenous WT UFBP1 or UFBP1 K267R in the livers of ND mice exerted no obvious effect on hepatic lipid accumulation or histopathologic changes.** **A** Mice aged 8 weeks were fed with normal diet (ND) for 12 weeks. Then Control AAV8, AAV8 expressing WT UFBP1 or AAV8 expressing UFBP1 K267R were injected via tail veins to infect hepatocytes of these mice (n=4 in each group). All these ND mice were sacrificed at 12 weeks post-AAV injection. **B** WB analysis of UFBP1 in the livers from the indicated groups at 12 weeks post-AAV injection. **C** Representative images of ORO and HE staining of liver sections from the indicated groups at 12 weeks post-AAV injection. ORO positive areas were quantified by calculating the ratio of the ORO stained area to the total area of an image using Image-Pro Plus and were normalized to those of the control group (n=4 in each group. Scale bar, 50 μm). The data in **C** were presented as the means ± SDs and analyzed by two- tailed Student’s t- test. n.s., non-specific signals.
