# Supplementary material for: Ufmylation on UFBP1 alleviates non-alcoholic fatty liver disease by modulating hepatic endoplasmic reticulum stress
Source: Cell Death Dis. 2023 Sep 2;14(9):584. doi: 10.1038/s41419-023-06095-2 (PMC10475044; doi:10.1038/s41419-023-06095-2)
Supplement: Supplementary file 2 — Supplementary Figure 2 [file 41419_2023_6095_MOESM2_ESM.docx]

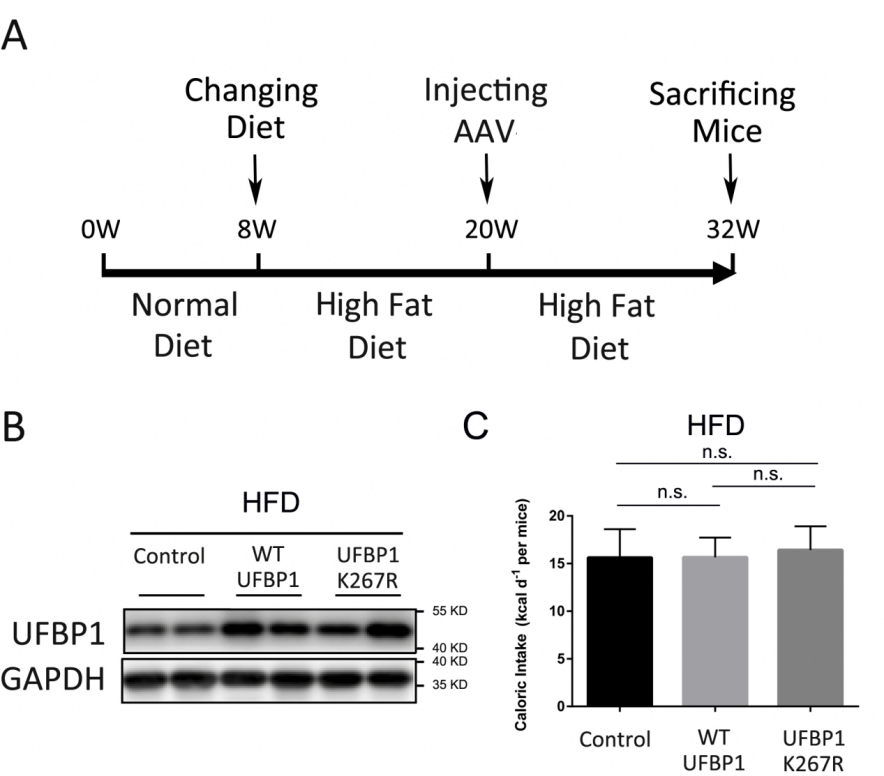


**Supplementary figure 2. Overexpressing exogenous WT UFBP1 or UFBP1 K267R in the livers of NAFLD mice exerted no obvious effect on caloric intake.** **A** Mice aged 8 weeks were fed with high fat diet (HFD) for 12 weeks to induce NAFLD. Then Control AAV8, AAV8 expressing WT UFBP1 or AAV8 expressing UFBP1 K267R were injected via tail veins to infect hepatocytes of NAFLD mice (n=4 in each group). All HFD mice were sacrificed at 12 weeks post-AAV injection. **B** WB analysis of UFBP1 in the livers from the indicated groups at 12 weeks post-AAV injection. **C** Caloric intake was calculated from food intake of mice in the control group, WT UFBP1 group and UFBP1 K267R group treated with HFD at 8 weeks post-AAV injection. The data in **C** were presented as the means ± SDs and analyzed by two- tailed Student’s t- test. n.s., non-specific signals.
