# Supplementary material for: Ufmylation on UFBP1 alleviates non-alcoholic fatty liver disease by modulating hepatic endoplasmic reticulum stress
Source: Cell Death Dis. 2023 Sep 2;14(9):584. doi: 10.1038/s41419-023-06095-2 (PMC10475044; doi:10.1038/s41419-023-06095-2)
Supplement: Supplementary file 3 — Supplementary table_1 [file 41419_2023_6095_MOESM3_ESM.docx]

**Supplementary** **table_1.**

**Primers** **for** **qPCR：**

| RT -h -UFM1 F | CAGTGTTCCTGAAAGTACACC  TT | RT -h -UFM1 R | CCGCAGTTCTGAACCATGTTT  TA |
| --- | --- | --- | --- |
| RT -h -UFBP1 F | AGGAGAGACCATGACTGAGG  A | RT -h -UFBP1 R | TCGTCAATCACACCTGTTATA  GT |
| RT -m -UFM1 F | GCTGCCGTACAAAGTTCTCAG | RT -m -UFM1 R | GTGCTTCAGGAAAACATTCC  CA |
| RT -m -UFBP1 F | TAGCGAAACCATGACTGAGG  A | RT -m -UFBP1 R | TCGTCAATCACACCTGTTAGA  GT |
| RT -h -GAPDH F | ACAACTTTGGTATCGTGGAAG  G | RT -h -GAPDH R | GCCATCACGCCACAGTTTC |
| RT -h - β-Actin F | AGAGCTACGAGCTGCCTGAC | RT -h - β-Actin R | AGCACTGTGTTGGCGTACAG |
| RT -m -GAPDH F | CCATGTTCGTCATGGGTGTGA  ACCA | RT -m -GAPDH R | GCCAGTAGAGGCAGGGATG  ATGTTC |
| RT -m - β Actin F | AACAGTCCGCCTAGAAGCAC | RT -m - β Actin R | CGTTGACATCCGTAAAGACC |
| RT -m -SREBP1 F | GCAGCCACCATCTAGCCTG | RT -m -SREBP1 R | CAGCAGTGAGTCTGCCTTGA  T |
| RT -m -SCD1 F | TCTTCCTTATCATTGCCAACAC  CA | RT -m -SCD1 R | GCGTTGAGCACCAGAGTGTA  TCG |
| RT -m -DGAT2 F | GCGCTACTTCCGAGACTACTT | RT -m -DGAT2 R | GGGCCTTATGCCAGGAAACT |
| RT -m -CD36 F | GCTTGCAACTGTCAGCACAT | RT -m -CD36 R | GCCTTGCTGTAGCCAAGAAC |
| RT -m -PPARγ F | TATCACTGGAGATCTCCGCCA  ACAGC | RT -m -PPARγ R | GTCACGTTCTGACAGGACTG  TGTGAC |
| RT -h -SREBP1 F | ACGGGAGGATGGACTGACTT | RT -h -SREBP1 R | AGGCTTCTTTGCTGTGAGATG |
| RT -h -SCD1 F | TTCCTACCTGCAAGTTCTACA  CC | RT -h -SCD1 R | CCGAGCTTTGTAAGAGCGGT |
| RT -h -DGAT2 F | ATCTTCTCTGTCACCTGGCT | RT -h -DGAT2 R | ACCTTTCTTGGGCGTGTTCC |
| RT -h -CD36 F | TCTGAAAGGAATCCCTGTGT | RT -h -CD36 R | TGCATTTGCTGATGTCTAGC |
| RT -h -PPARγ F | CCGGAGAACAATCAGATTGA  AG | RT -h -PPARγ R | AGCTGGTCGATATCACTGGA  G |
| RT -m -GRP78 F | ACTTGGGGACCACCTATTCCT | RT -m -GRP78 R | ATCGCCAATCAGACGCTCC |
| RT -m -XBP1s F | CTGAGTCCGAATCAGGTGCAG | RT -m -XBP1s R | GTCCATGGGAAGATGTTCTG  G |
| RT -m -Caspase 2 F | CACCCTCTTCAAGCTTTTGG | RT -m -Caspase 2 R | CGAAAACCTCTTGGAGCTG |
| RT -h -GRP78 F | CATCACGCCGTCCTATGTCG | RT -h -GRP78 R | CGTCAAAGACCGTGTTCTCG |
| RT -h -XBP1s F | AACCAGGAGTTAAGACAGCG  CTT | RT -h -XBP1s R | CTGCACCCTCTGCGGACT |
| RT -h -Caspase 2 F | CTATGTGACCAGACTGCACA | RT -h -Caspase 2 R | GTGCCACGATGCAGGAGTC |
