# Supplementary material for: Ufmylation on UFBP1 alleviates non-alcoholic fatty liver disease by modulating hepatic endoplasmic reticulum stress
Source: Cell Death Dis. 2023 Sep 2;14(9):584. doi: 10.1038/s41419-023-06095-2 (PMC10475044; doi:10.1038/s41419-023-06095-2)

Figure 1 D

UFM1


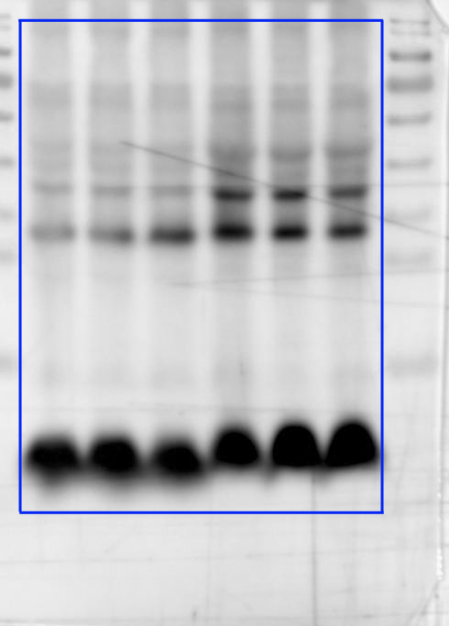


GAPDH


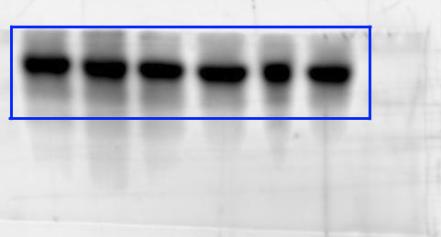


Figure 1 E

UFM1


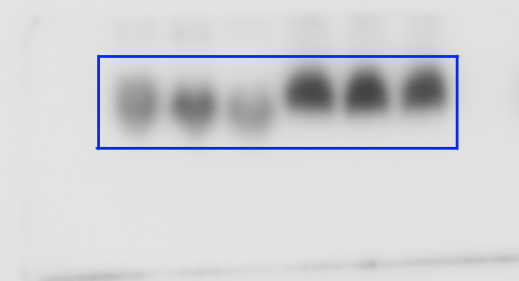


UFBP1


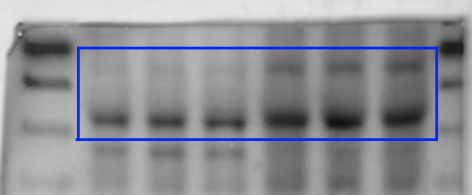


UBA5


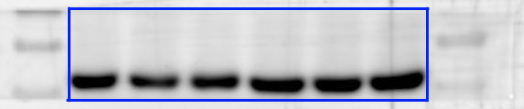


、

UFC1


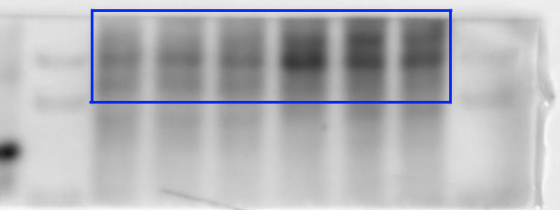


UFL1


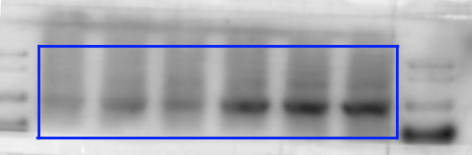


GAPDH


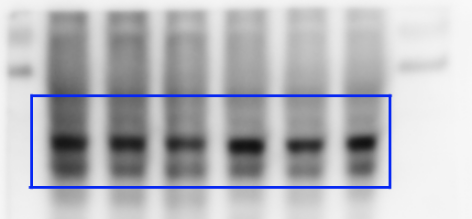


Figure 1 G

UFM1


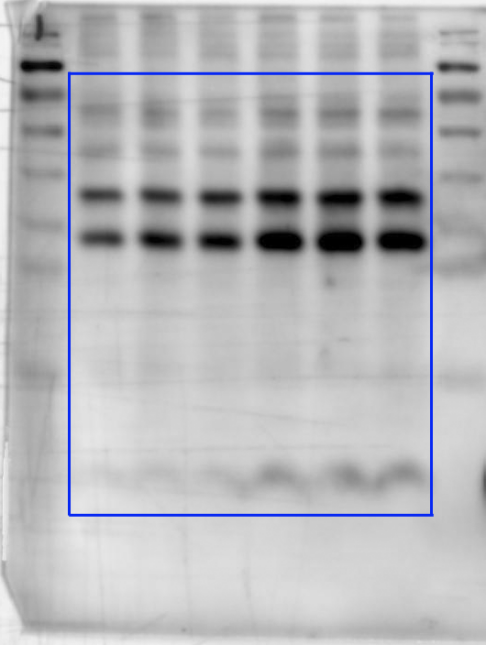


GAPDH


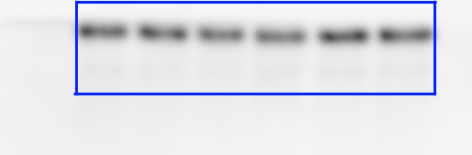


Figure 1 H

UFM1


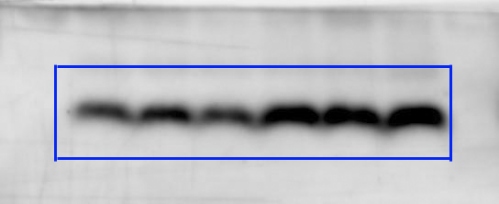


UFBP1


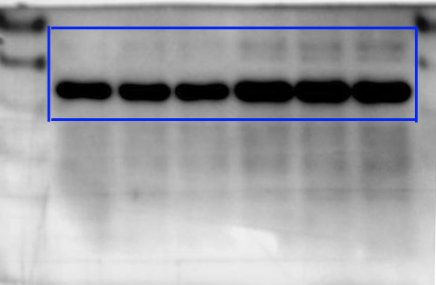


UBA5


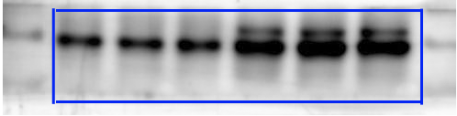


UFC1


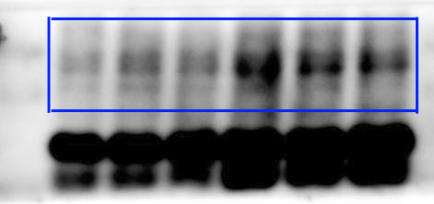


UFL1


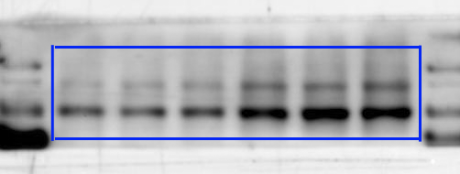


GAPDH


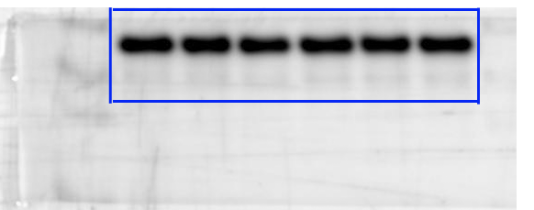


Figure 2 A

UFM1


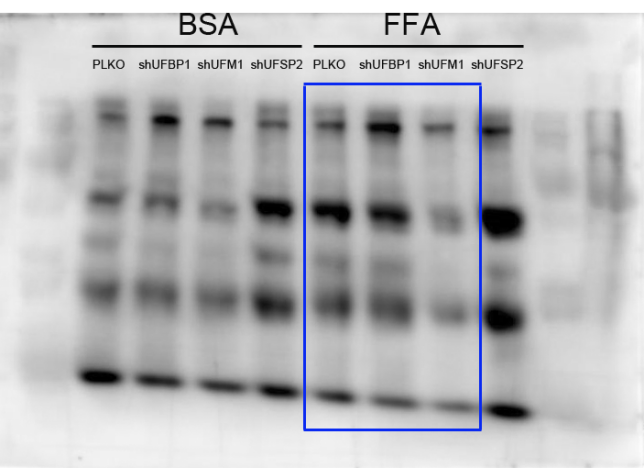


UFBP1


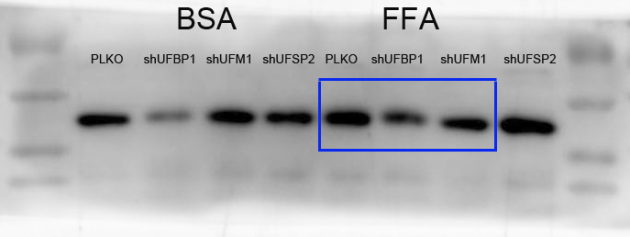


GAPDH


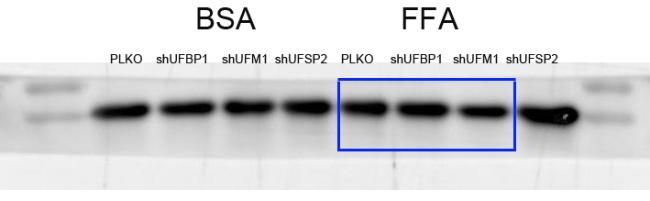


Figure 2 D

SREBP1


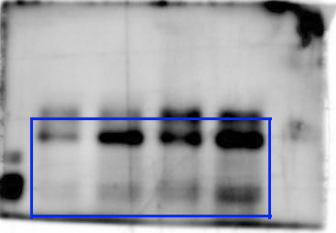


SCD1


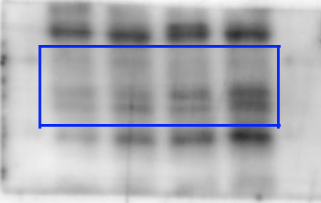


PPAR y


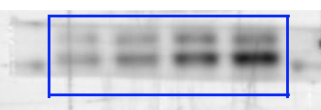


CD36


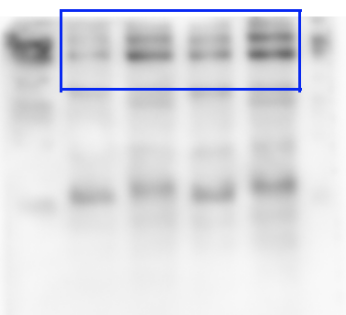


UFBP1


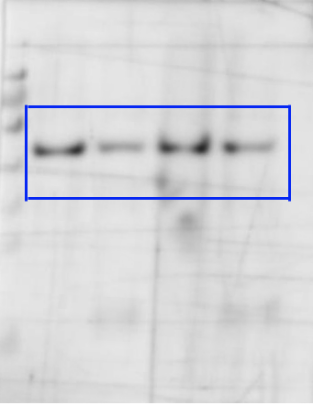


GAPDH


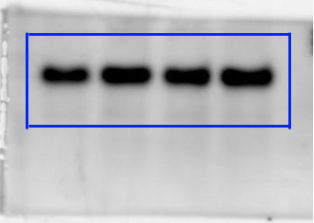


Figure 3 A

Input

UFM1


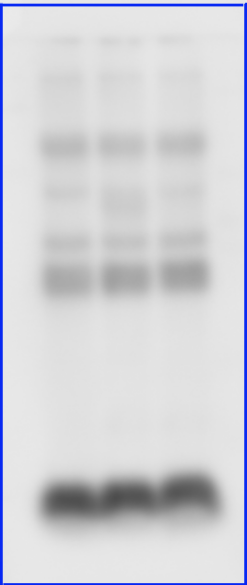


UFBP1


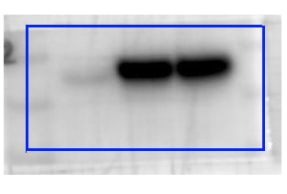


UBA5


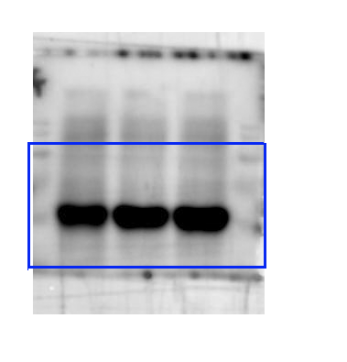


UFC1


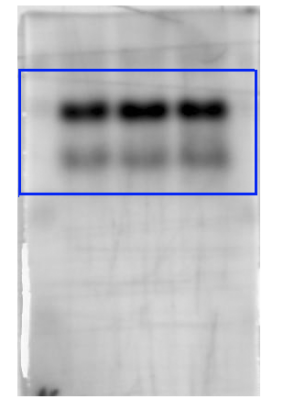


UFL1


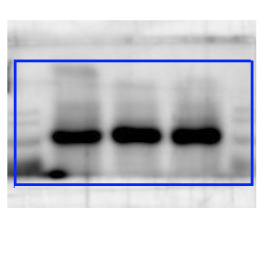


GAPDH


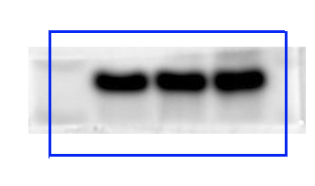


IP Flag

UFM1


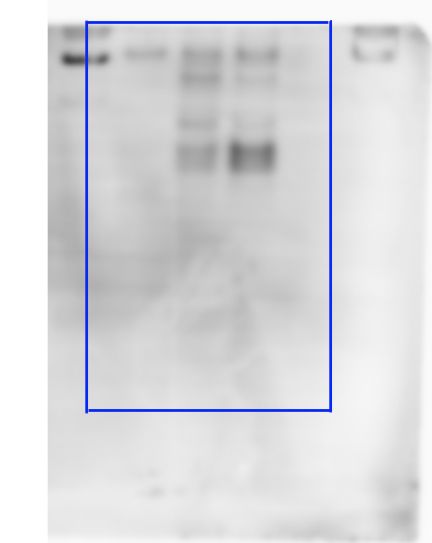


UFBP1


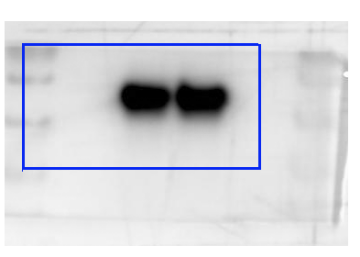


IP HA

UFM1


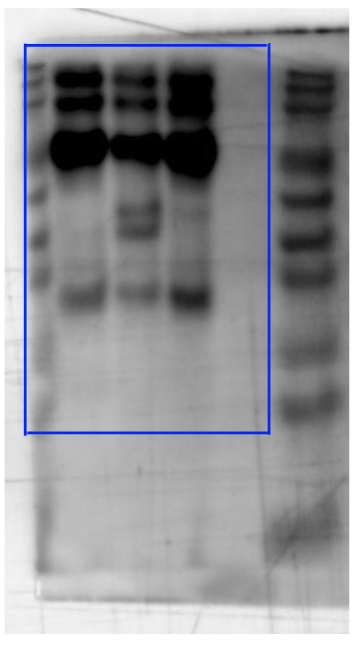


UFBP1


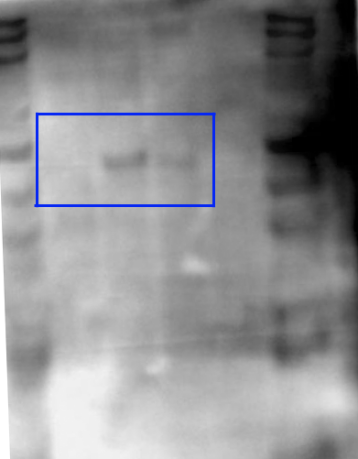


Figure 3 C

SREBP1


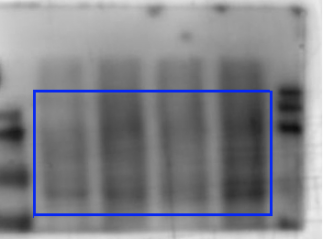


SCD1


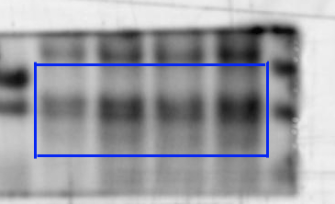


PPAR Y


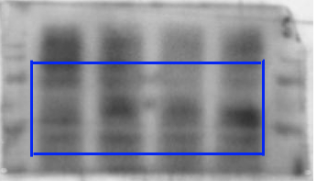


CD36


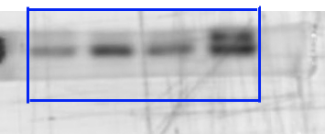


UFBP1


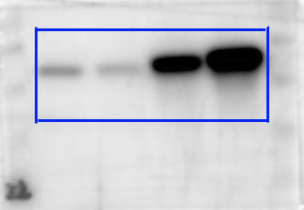


GAPDH


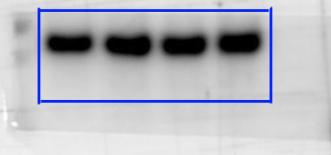


Figure 4 I

SREBP1 Precursor


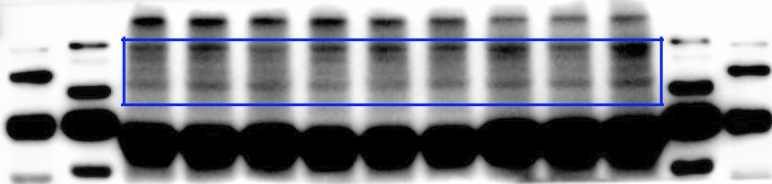


SREBP1 cleved


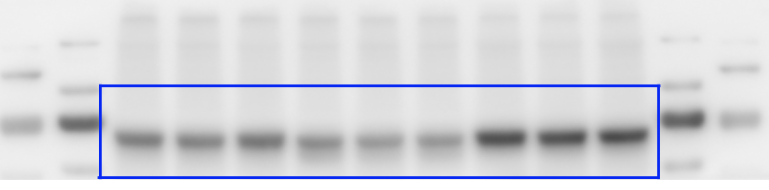


SCD1


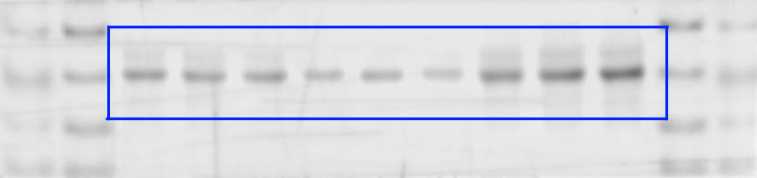


PPAR Y


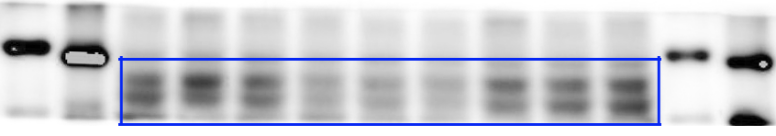


CD36


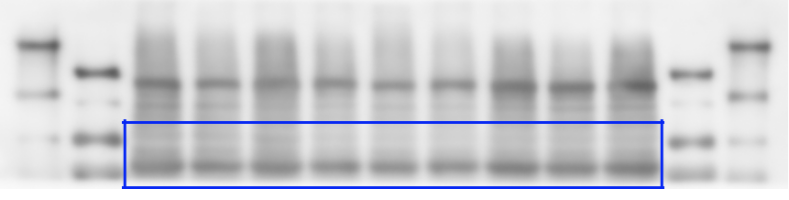


UFBP1


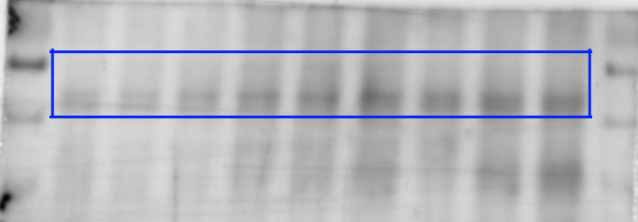


1. Actin


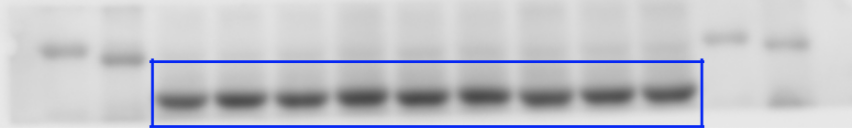


Figure 5 D

1. AKT


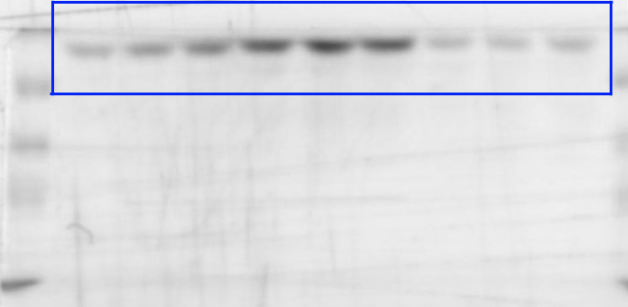


AKT


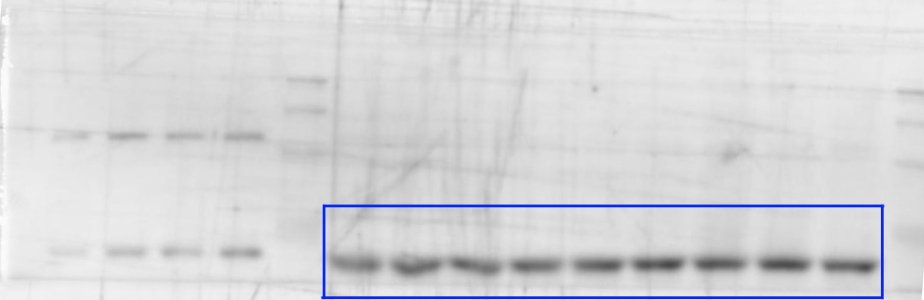


q-GSK3B


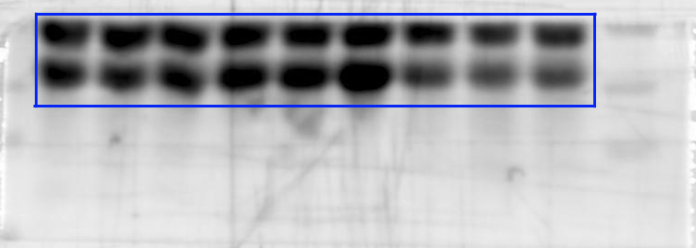


GSK3B


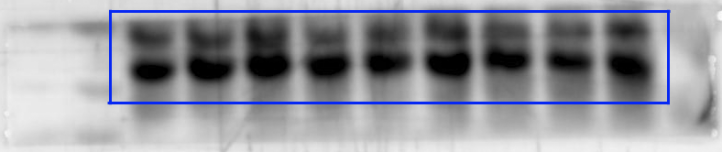


GAPDH


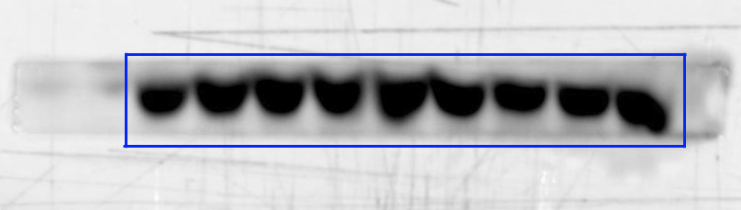


Figure 6 B

GRP78


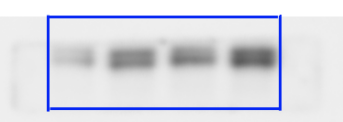


ATF4


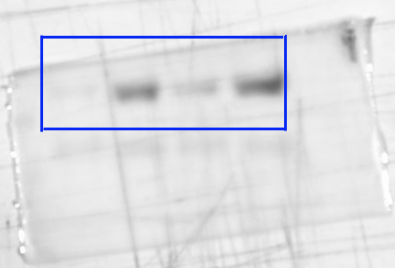


XBP1s


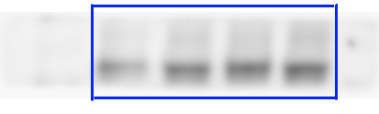


Caspase 2


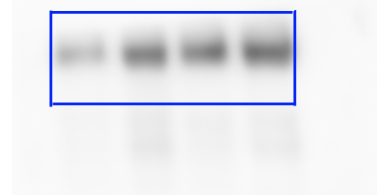


ATF6


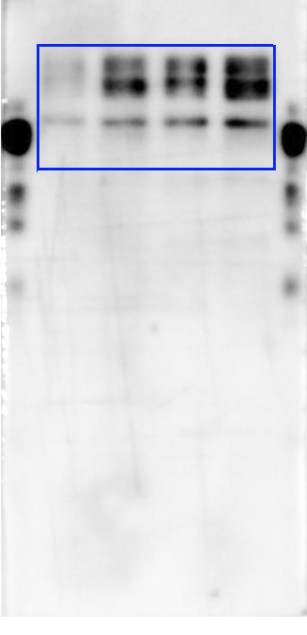


GAPDH


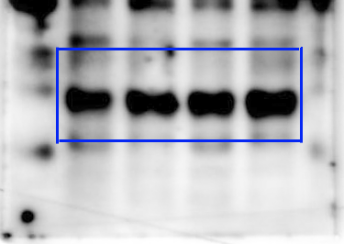


1. PERK


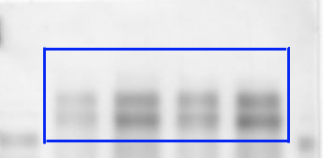


PERK


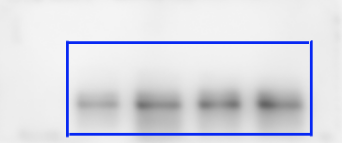


P-eIF2a


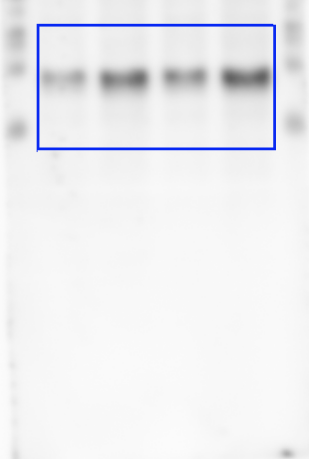


eIF2a


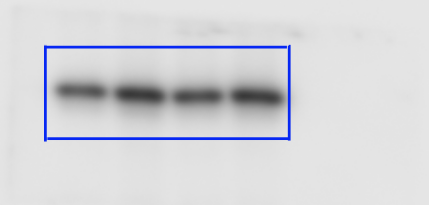


P-IRE1a


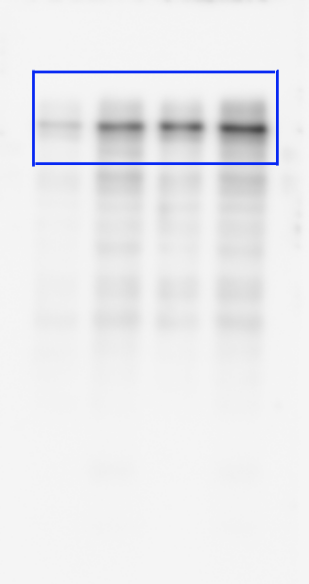


IRE1a


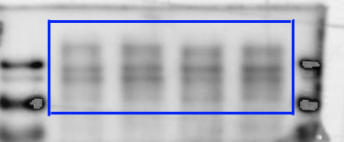


Figure 6 D

GRP78


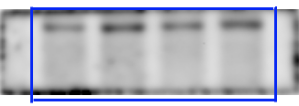


ATF4


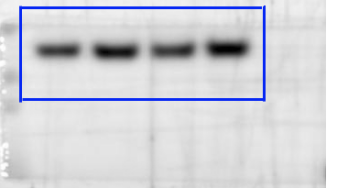


XBP1s


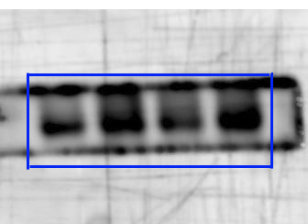


Caspase 2


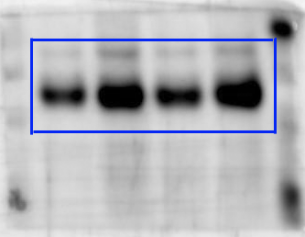


ATF6


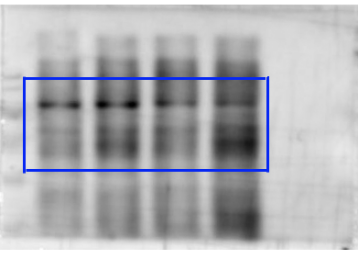


GAPDH


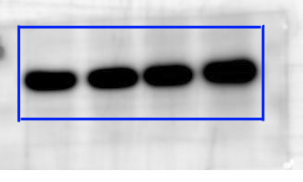


P-PERK


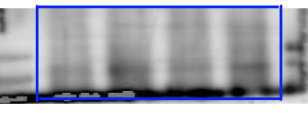


PERK


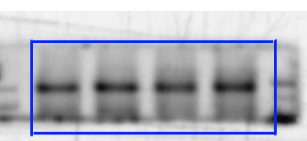


P-eIF2a


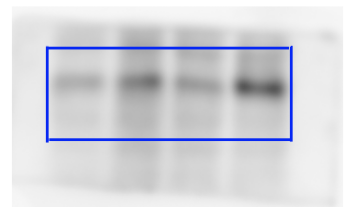


eIF2a


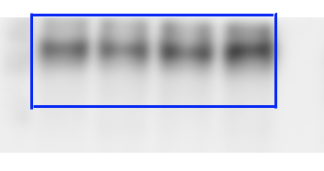


P-IRE1a


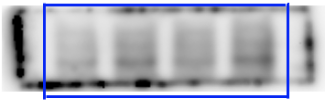


IRE1a


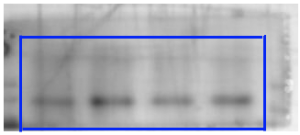


Figure 6 F

GRP78


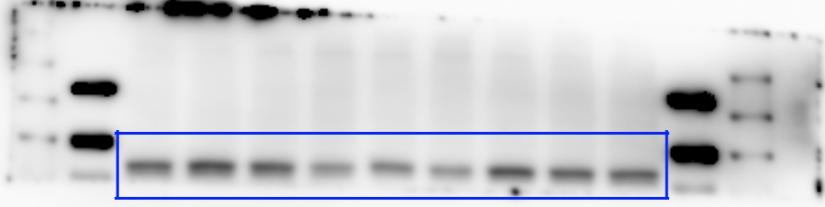


ATF4


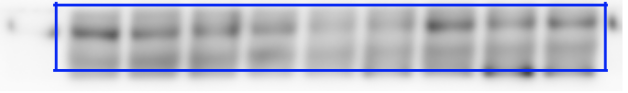


XBP1s


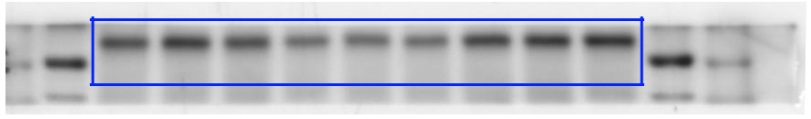


Caspase 2


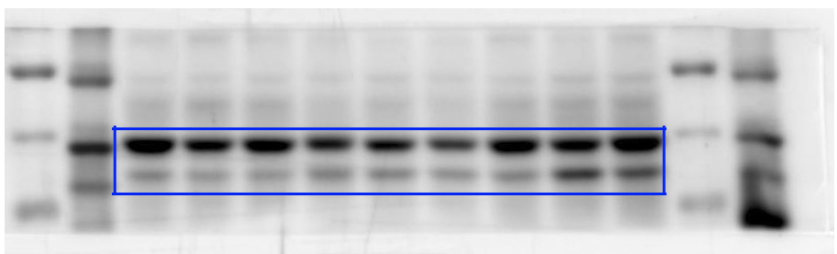


ATF6


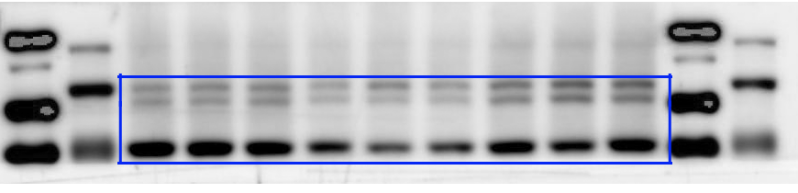


B-Actin


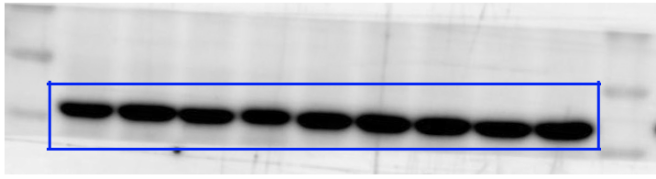


P-PERK


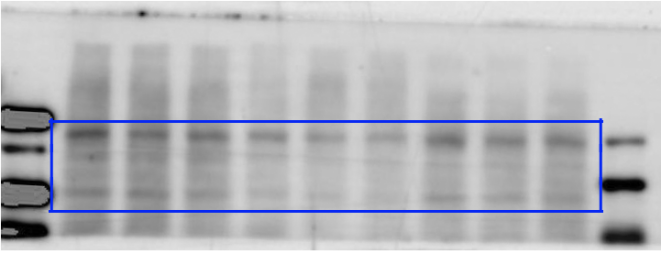


PERK


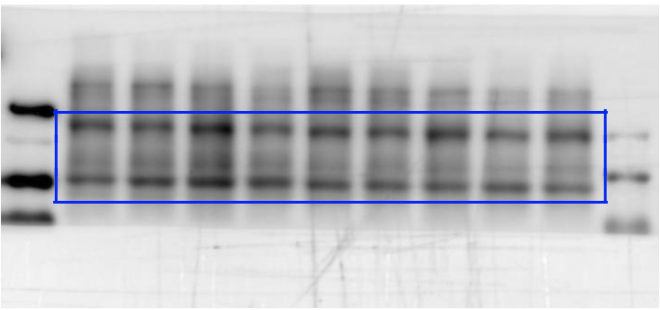


P-eIF2a


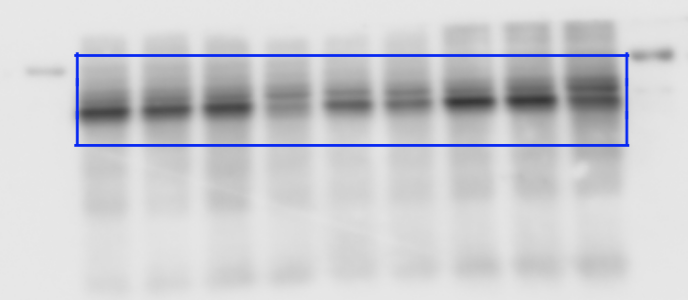


eIF2a


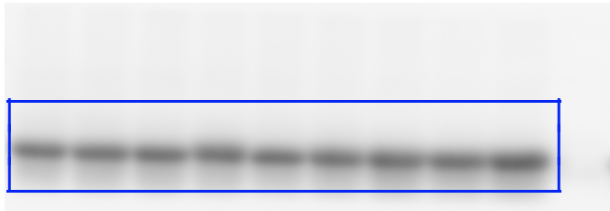


P-IRE1a


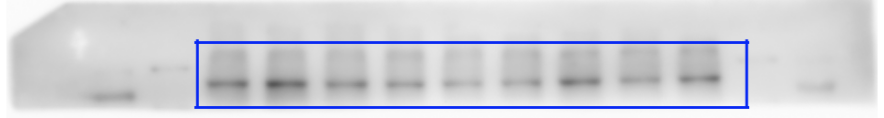


IRE1a


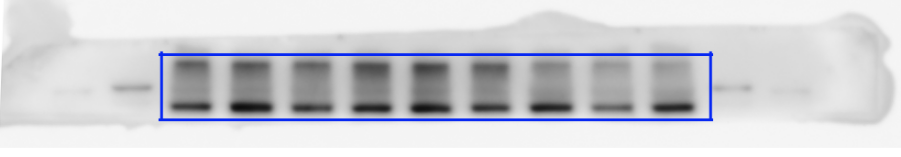


Supplementary figure_1

UFBP1


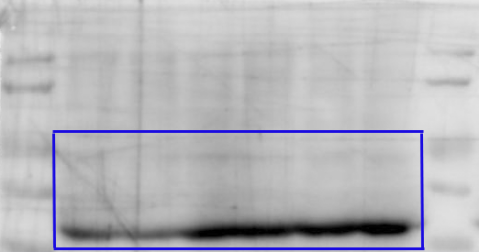


GAPDH


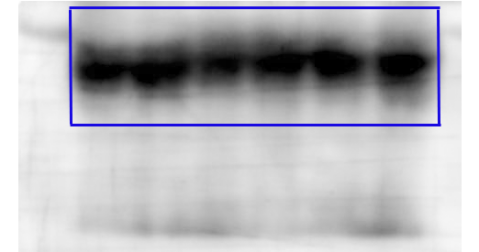

Supplement: Supplementary file 4 — Original western blots [file 41419_2023_6095_MOESM4_ESM.docx]
